# Sugar‐Sweetened Beverages, Artificially Sweetened Beverages and Sugar Forms With Long‐Term Risk of Irritable Bowel Syndrome: A Large‐Scale Prospective Cohort Study

**DOI:** 10.1002/fsn3.70094

**Published:** 2025-03-19

**Authors:** Shanshan Wu, Zhirong Yang, Si Liu, Qian Zhang, Shutian Zhang, Shengtao Zhu

**Affiliations:** ^1^ Department of Gastroenterology, Beijing Friendship Hospital Capital Medical University, National Clinical Research Center for Digestive Disease, Beijing Digestive Disease Center, Beijing Key Laboratory for Precancerous Lesion of Digestive Disease Beijing China; ^2^ Shenzhen Institute of Advanced Technology Chinese Academy of Sciences Shenzhen China; ^3^ Primary Care Unit, Department of Public Health and Primary Care, School of Clinical Medicine University of Cambridge Cambridge UK

**Keywords:** artificially sweetened beverages, irritable bowel syndrome, natural juice, sugar‐sweetened beverages

## Abstract

We aimed to examine the prospective association of sugar‐sweetened beverages (SSB), artificially sweetened beverages (ASB), natural juice, and sugar forms with irritable bowel syndrome (IBS). Participants free of IBS, celiac disease, inflammatory bowel disease, and any cancer at recruitment were included (*N* = 178,711, 53.1% female). SSB, ASB, natural juice, and different sugar forms' consumption were measured via a 24‐h dietary recall questionnaire. The primary outcome was incident IBS. A Cox proportional hazard model adjusting for age, sex, BMI, Townsend deprivation index, education, ethnicity, smoking, alcohol drinking, physical activity, total energy intake, type 2 diabetes, depression, and anxiety was conducted to assess the relationship. Mean consumption of SSB, ASB, and natural juice was 90.0, 72.4, and 105.7 g/day, respectively. During a median of 11.3‐year follow‐up, 2690 participants developed IBS. Every 100 g/day SSB increment was associated with a 3% higher IBS risk (HR = 1.03, 95% CI: 1.01–1.05). Compared with no SSB intake, the highest quartile was associated with an increased risk of IBS (HR = 1.19, 1.03–1.37; *p*_trend_ = 0.017). Regarding ASB and natural juice, no significant association was detected in those who consumed the highest quartile versus no intake (ASB: HR_Q4 VS no intake_ = 1.12, 0.95–1.31, *p*_trend_ = 0.062; Natural juice: HR_Q4 VS no intake_ = 1.01, 0.87–1.18, *p*_trend_ = 0.363). Considering different sugar forms, increased IBS risk was detected in added sugar (HR_Q4 VS Q1_ = 1.20, 1.05–1.36, *p*_trend_ = 0.001), instead of naturally occurring sugar (HR_Q4 VS Q1_ = 0.99, 0.88–1.11, *p*_trend_ = 0.869). Higher intake of SSB, rather than ASB and natural juice, is associated with increased IBS risk. Higher consumption of added sugar, instead of naturally occurring sugar, is associated with higher IBS risk. These findings highlight the importance of limiting SSB consumption in diets to reduce the modifiable burden of IBS.

## Graphical abstract

In this large‐scale prospective cohort, higher intake of sugar‐sweetened beverages is associated with a higher risk of incident IBS, rather than the null association for artificially sweetened beverages or natural juice intake. Higher consumption of added sugar, instead of naturally occurring sugar, is associated with an increased risk of IBS development, suggesting the potential role of sugar forms rather than simply the amount.

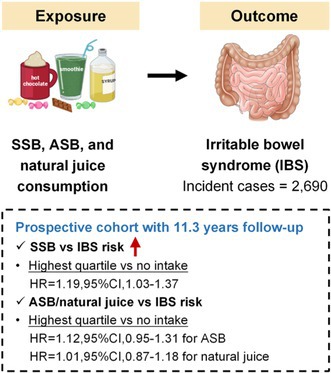

## 1 Introduction

Irritable bowel syndrome (IBS) is the most common gut‐brain interaction disorder, which is impaired communication between the gut and the brain via the nervous system in both directions (from gut to brain and brain to gut). It is reported that IBS affects 4.1% and 10.1% of adults worldwide according to Rome III and IV criteria (Sperber et al. 2021). It is characterized by recurrent abdominal pain, altered bowel habits, and bloating/distension owing to intestinal barrier damage and visceral hypersensitivity, accordingly resulting in substantial adverse impact on quality of life and significant economic burden (Lacy et al. 2021; Frändemark et al. 2018). Hence, it is of high priority to identify modifiable contributing factors to help develop targeted strategies on reducing IBS burden.

Interestingly, there is growing concern about the role of diet in the development and treatment of IBS. Several lines of evidence demonstrate that a diet high in fermentable oligosaccharides, disaccharides, monosaccharides, and polyols (FODMAPs), certain short‐chain carbohydrates that the small intestine absorbs poorly, may trigger or aggravate IBS symptoms via adverse impacts on gut motility, gut microbiota, and intestinal permeability (Lacy et al. 2021; McKenzie et al. 2016). Sugar‐sweetened beverages (SSB), artificially sweetened beverages (ASB) and natural juice, as the largest sources of FODMAPs in the diet, are consumed at a high level worldwide, accompanied by urbanization. Generally, SSBs are the largest source of added sugar, including soft drinks, fruit drinks, sports drinks, and energy drinks (Malik and Hu 2022). By contrast, pure natural juice is the major source of naturally occurring sugar, instead of added sugar (Malik and Hu 2022). ASBs are marketed as healthier options with low or no calories and suggested as alternatives to SSBs, containing artificial sweeteners such as aspartame, saccharin, and sucralose, etc. Emerging data suggest a possible link between dietary sugar and IBS through plausible mechanisms, including increased mucosal inflammation, gut microflora disturbance, impaired gut motility, and increased secretion of incretin hormones and serotonin, which may further induce intestinal barrier damage and visceral hypersensitivity and eventually lead to IBS development (Malik and Hu 2022; Spencer et al. 2016; Singh and Nee 2021). However, to the best of knowledge, there is a remarkable paucity of epidemiological evidence on the relationship between SSBs, ASBs, and natural juice consumption and the risk of incident IBS. Additionally, different types of beverages, as well as sugar forms, and their link with IBS occurrence remain to be answered yet.

To address these knowledge gaps, we prospectively examined the association of sugar‐sweetened beverages, artificially‐sweetened beverages, and natural juice intake with the risk of incident IBS in a large‐scale population‐based cohort with long‐term follow‐up, in order to provide more evidence for restricting their consumption and improving the overall health of the general population.

## 2 Materials and Methods

### 2.1 Study Population

This study was based on an ongoing large‐scale prospective cohort, UK Biobank (UKB), with over 500,000 participants from 22 assessment centers across England, Wales, and Scotland being enrolled between 2006 and 2010. All participants completed baseline questionnaires with anthropometric assessments and medical conditions reports. Over 210,000 participants finished a web‐based 24 h diet recall questionnaire (Oxford WebQ) in five cycles between April 2009 and June 2012 (Sudlow et al. 2015). The UKB study was approved by the North West Multicenter Research Ethical Committee(21/NW/0157), and all participants or their proxy respondents provided written informed consent.

Participants who were free of IBS before the 24 h diet recall questionnaire with available SSB, ASB, and natural juice intake information were included. Those who had a previous cancer, inflammatory bowel disease (IBD) or celiac disease diagnosis prior to baseline were excluded. All diagnoses were identified through International Classification of Disease‐10th revision (ICD‐10) codes. In order to ensure data accuracy and reduce reporting bias, those with total energy intake > 5000 kcal or < 500 kcal (i.e., implausibly high or low energy intake) were excluded. Additionally, 10 participants who withdrew from the study was excluded. Therefore, a total of 178,711 participants (53.1% female, mean [SD] age of 55.8 [8.0] years) were included (Figure S1), which was relatively older than the whole general population in the UK.

### 2.2 Assessment of Baseline SSB, ASB, Natural Juice, and Different Forms of Sugar Intake

Dietary information was collected via Oxford WebQ from five cycles (cycle 1: April 2009–September 2010, cycle 2: February 2011–April 2011, cycle 3: June 2011–August 2011, cycle 4: October 2011–December 2011, cycle 5: April 2012–June 2012), in order to maximize the coverage of all seasons and days of the week. Participants were asked how many units (glasses/cans/250 mL/cartons) of beverages they drank yesterday. Beverages' intake (g/day) was generated by multiplying the number of portions consumed by the set quantity of each portion size (Piernas et al. 2021). SSB referred to fizzy sugary drinks, squash, and fruit smoothies. ASB included low‐calorie fizzy drinks, whereas natural juice referred to pure orange juice, grapefruit juice, and other 100% fruit juice. In order to ensure accuracy, an average value of SSB, ASB, and natural juice intake as well as total energy was calculated for participants who completed ≥ 2 dietary cycles (arithmetic mean value of all dietary cycles). To examine the association of SSB, ASB, and natural juice intake with incident IBS, quartiles of each beverage intake for those who consumed > 0 g/day were defined, with no intake of this beverage as the reference group. Meanwhile, the continuous variable (i.e., per 100 g increment of SSB, ASB, and natural juice intake) was also considered.

Additionally, total sugar and different forms of sugar intake (added sugar or naturally occurring sugar) were measured from all types of food intake based on the 24‐h diet recall Oxford WebQ questionnaire, including both three types of beverages (SSB, ASB, and natural juice) and all other 206 types of food. Details of each type of food and beverages were described in Perez‐Cornago et al. 2021, using the food composition table from UK Nutrient Databank (UKNDB) (Perez‐Cornago et al. 2021). Naturally occurring sugar was defined as intrinsic and milk sugar, whereas added sugar was defined as the difference between total sugar and naturally occurring sugar. Since SSB/ASB and natural juice were the major sources of added sugar and naturally occurring sugar, the estimated total sugar and different forms of sugar intake (g/day) can also indirectly reflect the consumption of overall beverages, SSB/ASB, and natural juice. Hence, we considered this as a sensitivity analysis to verify the robustness of our results, with total sugar and different forms of sugar intake as additional exposures. The risk of incident IBS associated with total sugar intake, added sugar intake, and naturally occurring sugar intake was assessed separately by adjusting multiple covariates in the following.

### 2.3 Ascertainment of Incident IBS

Primary endpoint was incident IBS, which was determined via ICD‐10 codes (K58). ICD‐10 code (International Classification of Diseases, Tenth Revision) is a standardized system used to code diseases and medical conditions (morbidity) data, which is owned and published by the World Health Organization (WHO). IBS diagnosis was ascertained based on linkage to primary care and/or hospital admission data obtained from Hospital Episode Statistics for England, Scottish Morbidity Record data for Scotland, and Patient Episode Database for Wales. The censoring date (i.e., the end follow‐up date of the study) was set on May 30, 2022.

### 2.4 Covariates

Covariates were considered based on previous epidemiological evidence, including age (continuous variable), sex (male or female), BMI (underweight, normal, overweight, obese), ethnicity (white or nonwhite), Townsend deprivation index (TDI, quartiles), education level (university or non‐university), smoking status (never, current, previous), alcohol drinking (never, current, or previous), type 2 diabetes (Yes or No), depression (Yes or No), anxiety (Yes or No), physical activity, diet pattern, and total energy intake (continuous) (Lacy et al. 2021; McKenzie et al. 2016; Malik and Hu 2022). All covariates except total energy intake were collected at baseline (2006–2010) through touch‐screen questionnaires with anthropometric assessments and medical conditions reports. We assumed those covariates were unchanged from baseline (2006–2010) to the Oxford WebQ dietary assessment (February 2011–June 2012). TDI was a measure of socioeconomic status, with a lower value indicating relative affluence. Baseline comorbidity was identified via ICD‐10 code (E11 for type 2 diabetes, F32 for depression, F40 and F41 for anxiety) through linkage to primary care and/or hospital admission data. Self‐reported physical activity was divided into three levels (high, moderate, low) based on IPAQ (International Physical Activity Questionnaire). Diet pattern was defined based on the following 7 dietary components ([1] fruits ≥ 3 servings/day; [2] vegetables ≥ 3 servings/day; [3] fish ≥ 2 servings/week; [4] processed meats ≤ 1 serving/week; [5] unprocessed red meats ≤ 1.5 servings/week; [6] whole grains ≥ 3 servings/day; [7] refined grains ≤ 1.5 servings/day) (Mozaffarian 2016). Participants who fulfilled each of the above 7 items would achieve 1 healthy diet score. Therefore, the total diet score would range from 0 to 7. A healthy diet pattern was defined as a diet score ≥ 4. Total energy intake was obtained from a web‐based 24‐h dietary recall questionnaire.

### 2.5 Statistical Analysis

The cumulative incidence of IBS was calculated by the Kaplan–Meier method. Due to the longitudinal time‐to‐event data obtained during a long‐term follow‐up period, the Cox proportional hazard model, the most popular model for the analysis of time‐to‐event data in the cohort design, was performed to examine the associated risk of incident IBS with SSB, ASB, and natural juice consumption, respectively. The proportional hazard assumption was ascertained and satisfied by testing linear regression of scaled Schoenfeld residuals on functions of time (*p* = 0.120, 0.220, 0.790 for SSB, ASB and natural juice intake, separately) (Hess 1995). The follow‐up period started from the date of the 24‐h dietary recall questionnaire to the date of the first IBS diagnosis or was censored at the end of the study (May 30, 2022), the date of death, or losttofollow‐up for participants who did not develop IBS. The median follow‐up was 11.3 years for the whole cohort. Considering the very small percentage of missing values (0.1%–0.5%) for most variables, missing indicators were used.

For quartiles and per 100 g/day increment of SSB, ASB, and natural juice consumption, three models were conducted: (1) model 1, adjusted for age and sex; (2) model 2, additionally adjusted for BMI, TDI, education level, ethnicity, smoking status, and alcohol drinking; (3) model 3, additionally adjusted for IPAQ, total energy intake, type 2 diabetes, depression, and anxiety. Furthermore, subgroup analysis was conducted to assess whether the association varied by age (< 60, ≥ 60 years), sex, BMI (≤ 25, > 25 kg/m^2^), smoking status (never, current/previous), alcohol drinking (never/previous, current) and diet pattern (unhealthy, healthy). Effect modification was detected by adding interaction terms for each stratified variable and SSB/ASB/natural juice consumption. Trend analysis was performed by using median values of each quartile for SSB/ASB/natural juice.

Furthermore, the association of total sugar, added sugar, as well as naturally occurring sugar intake and incident IBS risk was also examined via model 3, with the lowest quartile intake as the reference group. Moreover, IBS risk associated with a per 10 g/day increment of total sugar, added sugar, and naturally occurring sugar intake was investigated. Similarly, subgroup analyses by age (< 60, ≥ 60 years) and sex, as well as effect modification and trend analyses were further conducted.

Additional sensitivity analyses were conducted. Firstly, participants who had an IBS diagnosis within 1 or 2 years after the 24‐h dietary recall questionnaire were excluded respectively, in order to avoid reverse causation. Secondly, SSB and natural juice were mutually adjusted to further control for confounding (i.e., additionally adjusting natural juice when assessing the risk of IBS associated with SSB, and additionally adjusting SSB when assessing IBS risk associated with natural juice). Thirdly, to rule out the mediation effect of total energy intake, models were re‐run without adjusting total energy intake as a covariate. Fourthly, a competing risk model considering lost‐to‐follow‐up and death as competing events was conducted, since those participants might develop IBS thereafter. Fifthly, the diet pattern was additionally adjusted to further rule out the influence of the diet pattern. Sixthly, considering different energy intake for males and females, we further excluded participants with total energy intake > 4200 kcal or < 800 kcal for males and > 3500 kcal or < 500 kcal for females. Finally, quintiles of SSB, ASB, and natural juice were used in the sensitivity analyses in addition to quartiles of those beverages to further verify the robustness of the results.

A two‐tailed *p* value < 0.05 was considered to be statistically significant. All analyses were conducted using SAS software Version 9.4 and R version 4.0.2 (forestplot, tableone, ggplot2, and survival packages).

### 2.6 Patient and Public Involvement

No patients and the public were involved in the design, conduct, reporting, and dissemination plans of this research.

## 3 Results

### 3.1 Baseline Characteristics

Overall, 63,257 (35.4%), 36,483 (20.4%), and 92,456 (51.7%) participants consumed SSB, ASB, and natural juice, respectively. SSB drinkers were more likely to be male, never/previous drinkers, had a lower level of education and socioeconomic deprivation, a higher BMI, higher total sugars, total energy intake, and ASB intake (Table 1). Regarding ASB consumption, ASB drinkers were more likely to be female, had a higher level of socioeconomic deprivation and BMI, a higher proportion of prevalent diabetes and depression, a lower alcohol, total sugars, total energy intake, and natural juice intake (Table S1). Natural juice consumers were more likely to be male, never smokers, had a higher level of education and socioeconomic deprivation, a lower BMI, a lower proportion of prevalent diabetes and depression, a higher total sugars and total energy intake, and a lower ASB intake (Table S2). The mean consumption of SSB, ASB, and natural juice was 90.0, 72.4, and 105.7 g/day, respectively. A total of 2690 incident IBS cases developed during 1,988,347 person‐years of follow‐up (median: 11.3 years; interquartile range: 10.8–12.1 years).

**TABLE 1:** Baseline characteristics according to baseline sugar‐sweetened beverages consumption in the cohort.

Characteristic	Total (*N* = 178,711)	0 (*N* = 115,454)	Quartile 1 (*N* = 16,925)	Quartile 2 (*N* = 14,578)	Quartile 3 (*N* = 19,181)	Quartile 4 (*N* = 12,573)
Age (years)^a^	55.81 ± 7.96	56.30 ± 7.83	56.10 ± 7.76	55.40 ± 8.04	54.63 ± 8.19	53.12 ± 8.20
Sex						
Male	83,731 (46.9)	52,326 (45.3)	7533 (44.5)	6980 (47.9)	9761 (50.9)	7131 (50.7)
Female	94,980 (53.1)	63,128 (54.7)	9392 (55.5)	7598 (52.1)	9420 (49.1)	5442 (49.3)
Nutrient and food intake						
Total energy intake (KJ/day)^a^	8648 ± 2447	8425 ± 2422	8671 ± 2020	8792 ± 2296	9110 ± 2535	9786 ± 2779
Total daily intake (g/day)^a^, ^b^	3221 ± 799	3177 ± 786	3167 ± 675	3207 ± 750	3304 ± 821	3588 ± 972
Protein (g/day)^a^	80.9 ± 24.5	80.2 ± 24.7	80.8 ± 19.7	81.1 ± 22.9	82.2 ± 25.5	85.0 ± 28.4
Fat (g/day)^a^	73.1 ± 28.4	71.6 ± 28.5	74.3 ± 23.8	74.6 ± 26.8	76.2 ± 29.7	79.3 ± 31.8
Carbohydrate (g/day)^a^	254.3 ± 78.5	243.3 ± 76.0	253.8 ± 63.4	262.0 ± 71.9	277.7 ± 79.7	312.0 ± 90.6
Englyst fiber (g/day)^a^, ^c^	17.8 ± 6.7	17.8 ± 6.8	17.8 ± 5.6	17.7 ± 6.3	17.7 ± 6.8	17.8 ± 7.3
Alcohol intake (g/day)^a^	17.3 ± 22.2	18.1 ± 23.0	16.9 ± 19.0	15.9 ± 20.1	15.5 ± 21.1	14.4 ± 22.0
Sugar‐sweetened beverages (g/day)^a^	90.0 ± 181.5	0.0 ± 0.0	73.4 ± 22.2	143.6 ± 19.2	265.0 ± 44.6	609.4 ± 263.7
Artificially sweetened beverages (g/day)^a^	72.4 ± 198.3	68.6 ± 202.4	59.8 ± 144.4	76.4 ± 177.3	80.4 ± 190.2	107.7 ± 247.6
Natural juice (g/day)^a^	105.7 ± 144.5	103.0 ± 145.3	112.0 ± 119.4	107.5 ± 131.0	107.8 ± 139.3	115.9 ± 185.2
Total sugars intake (g/day)^a^	124.8 ± 48.7	115.9 ± 45.5	124.2 ± 38.5	130.4 ± 43.4	142.6 ± 47.7	172.9 ± 59.6
Ethnicity						
Non‐White	8199 (4.6)	4655 (4.0)	610 (3.6)	773 (5.3)	1217 (6.3)	944 (7.5)
White	169,930 (95.1)	110,439 (95.7)	16,257 (96.1)	13,759 (94.4)	17,895 (93.3)	11,580 (92.1)
Unknown	582 (0.3)	360 (0.3)	58 (0.3)	46 (0.3)	69 (0.4)	49 (0.4)
Education level						
Non‐university	100,832 (56.4)	65,107 (56.4)	8630 (51.0)	8204 (56.3)	11,206 (58.4)	7685 (61.1)
University	77,000 (43.1)	49,767 (43.1)	8234 (48.6)	6313 (43.3)	7863 (41.0)	4823 (38.4)
Unknown	879 (0.5)	580 (0.5)	61 (0.4)	61 (0.4)	112 (0.6)	65 (0.5)
Townsend deprivation index						
Mean (SD)	−1.55 (2.88)	−1.59 (2.86)	−1.65 (2.81)	−1.63 (2.83)	−1.43 (2.98)	−1.21 (3.07)
Q1 (≤ −3.71)	44,592 (25.0)	29,041 (25.2)	4339 (25.6)	3720 (25.5)	4661 (24.3)	2831 (22.5)
Q2 (−3.71 to −2.30)	44,659 (25.0)	29,099 (25.2)	4262 (25.2)	3651 (25.0)	4687 (24.4)	2960 (23.5)
Q3 (−2.30 to 0.11)	44,618 (25.0)	28,885 (25.0)	4248 (25.1)	3686 (25.3)	4752 (24.8)	3047 (24.2)
Q4 (> 0.11)	44,614 (25.0)	28,293 (24.5)	4065 (24.0)	3502 (24.0)	5050 (26.3)	3704 (29.5)
Unknown	228 (0.1)	136 (0.1)	11 (0.1)	19 (0.1)	31 (0.2)	31 (0.2)
Smoking status						
Never	101,498 (56.8)	64,398 (55.8)	9985 (59.0)	8654 (59.4)	11,233 (58.6)	7228 (57.5)
Previous	62,545 (35.0)	41,528 (36.0)	5824 (34.4)	4837 (33.2)	6336 (33.0)	4020 (32.0)
Current	14,191 (7.9)	9234 (8.0)	1081 (6.4)	1037 (7.1)	1555 (8.1)	1284 (10.2)
Unknown	477 (0.3)	294 (0.3)	35 (0.2)	50 (0.3)	57 (0.3)	41 (0.3)
Alcohol drinking						
Never	5741 (3.2)	3340 (2.9)	497 (2.9)	504 (3.5)	804 (4.2)	596 (4.7)
Previous	5221 (2.9)	3088 (2.7)	467 (2.8)	428 (2.9)	659 (3.4)	579 (4.6)
Current	167,571 (93.8)	108,915 (94.3)	15,947 (94.2)	13,628 (93.5)	17,698 (92.3)	11,383 (90.5)
Unknown	178 (0.1)	111 (0.1)	14 (0.1)	18 (0.1)	20 (0.1)	15 (0.1)
IPAQ						
Low	27,668 (15.5)	17,667 (15.3)	2632 (15.6)	2285 (15.7)	2989 (15.6)	2095 (16.7)
Moderate	64,193 (35.9)	41,589 (36.0)	6364 (37.6)	5247 (36.0)	6846 (35.7)	4147 (33.0)
High	60,110 (33.6)	38,618 (33.4)	5574 (32.9)	4921 (33.8)	6509 (33.9)	4488 (35.7)
Unknown	26,740 (15.0)	17,580 (15.2)	2355 (13.9)	2125 (14.6)	2837 (14.8)	1843 (14.7)
BMI						
< 18.5 kg/m^2^	915 (0.5)	645 (0.6)	95 (0.6)	60 (0.4)	74 (0.4)	41 (0.3)
18.5–24.9 kg/m^2^	63,104 (35.3)	41,540 (36.0)	6605 (39.0)	5028 (34.5)	6210 (32.4)	3721 (29.6)
25.0–29.9 kg/m^2^	75,429 (42.2)	48,647 (42.1)	6960 (41.1)	6301 (43.2)	8235 (42.9)	5286 (42.0)
≥ 30 kg/m^2^	38,765 (21.7)	24,297 (21.0)	3224 (19.0)	3154 (21.6)	4606 (24.0)	3484 (27.7)
Unknown	498 (0.3)	325 (0.3)	41 (0.2)	35 (0.2)	56 (0.3)	41 (0.3)
Type 2 Diabetes	3498 (2.0)	2408 (2.1)	260 (1.5)	243 (1.7)	323 (1.7)	264 (2.1)
Depression	12,297 (6.9)	7810 (6.8)	1102 (6.5)	935 (6.4)	1376 (7.2)	1078 (8.6)
Anxiety	5581 (3.1)	3555 (3.1)	507 (3.0)	474 (3.3)	597 (3.1)	448 (3.6)

*Note:* Numbers are *n* (%) unless otherwise stated.

Abbreviations: BMI, body mass index; IPAQ, International Physical Activity Questionnaire.

^a^ Displayed as mean ± standard deviation.

^b^ Estimated intake of total food weight based on food and beverage consumption yesterday.

^c^ Estimated intake of dietary fiber using the Englyst method.

### 3.2 SSB Intake and Risk of Incident IBS

The 11‐year cumulative incidence of IBS was 1.7% (95% CI: 1.4%–1.9%) in the highest quartile group of SSB intake versus 1.5% (95% CI: 1.4%–1.5%) in no SSB drinkers. Compared with no SSB intake, the highest SSB quartile was associated with a significantly increased risk of IBS (HR = 1.19, 95% CI: 1.03–1.37; *p*_trend_ = 0.017) according to the fully adjusted model 3 (Table 2, Table S3). Moreover, every 100 g/day increment of SSB was associated with a 3% higher risk of IBS (HR = 1.03, 95% CI: 1.01–1.05).

**TABLE 2:** Risk of IBS associated with baseline sugar‐sweetened beverages, artificially sweetened beverages, and natural juice consumption.

	Sugar‐sweetened, artificially sweetened beverages/natural juice consumption, quartiles						100 g/day increment	*p*_trend_
	0	Quartile 1	Quartile 2	Quartile 3	Quartile 4	*p*_trend_		
Sugar‐sweetened beverages								
No. of participants	115,454	16,925	14,578	19,181	12,573		178,711	
No. of incident IBS	1697	265	225	291	212		2690	
Follow‐up, person‐years	1,282,797	190,553	162,134	213,406	139,458		1,988,348	
Adjusted model 1	Reference	1.05 (0.92–1.20)	1.07 (0.93–1.22)	1.07 (0.94–1.21)	1.24 (1.07–1.43)	0.003	1.03 (1.01–1.05)	0.001
Adjusted model 2	Reference	1.07 (0.93–1.22)	1.07 (0.93–1.23)	1.05 (0.93–1.19)	1.19 (1.03–1.37)	0.017	1.03 (1.01–1.05)	0.009
Adjusted model 3	Reference	1.08 (0.95–1.23)	1.08 (0.94–1.24)	1.06 (0.94–1.20)	1.19 (1.03–1.37)	0.017	1.03 (1.01–1.05)	0.010
Artificially sweetened beverages								
No. of participants	141,688	8659	9401	9322	9641		178,711	
No. of incident IBS	2059	133	153	167	178		2690	
Follow‐up, person‐years	1,576,106	97,695	104,774	103,020	106,752		1,988,348	
Adjusted model 1	Reference	1.02 (0.86–1.22)	1.10 (0.93–1.29)	1.20 (1.02–1.40)	1.24 (1.07–1.45)	0.001	1.03 (1.01–1.05)	< 0.001
Adjusted model 2	Reference	1.02 (0.85–1.21)	1.08 (0.91–1.27)	1.15 (0.98–1.35)	1.16 (0.99–1.36)	0.018	1.02 (1.00–1.04)	0.015
Adjusted model 3	Reference	1.01 (0.85–1.21)	1.07 (0.91–1.26)	1.14 (0.97–1.33)	1.12 (0.95–1.31)	0.062	1.02 (1.00–1.03)	0.055
Natural juice								
No. of participants	86,255	18,204	32,018	29,198	13,036		178,711	
No. of incident IBS	1384	285	452	383	186		2690	
Follow‐up, person‐years	957,877	204,999	355,413	324,456	145,603		1,988,348	
Adjusted model 1	Reference	0.96 (0.84–1.09)	0.90 (0.81–1.01)	0.84 (0.75–0.95)	0.94 (0.80–1.09)	0.017	0.98 (0.95–1.01)	0.160
Adjusted model 2	Reference	1.00 (0.88–1.14)	0.95 (0.85–1.06)	0.89 (0.79–1.00)	0.99 (0.85–1.16)	0.193	0.99 (0.97–1.02)	0.659
Adjusted model 3	Reference	1.01 (0.89–1.15)	0.97 (0.87–1.08)	0.91 (0.81–1.02)	1.01 (0.87–1.18)	0.363	1.00 (0.97–1.03)	0.951

*Note:* Values are hazard ratios (95% confidence intervals) unless stated otherwise. Adjusted model 1: Age and sex were adjusted; Adjusted model 2: BMI, Townsend deprivation index, education level, ethnicity, smoking status, and alcohol drinking were additionally adjusted; Adjusted model 3: IPAQ (International Physical Activity Questionnaire), total energy intake, type 2 diabetes, depression, and anxiety were additionally adjusted; *p* for trend was calculated by using the median value (82.5, 130, 250, and 500 g/day) of each sugar‐sweetened beverages quartile, the median value (82.5, 165, 330, and 660 g/day) of each artificially sweetened beverages quartile, and the median value (62.5, 125, 250, and 417 g/day) of each natural juice quartile. *p* for trend was calculated by using continuous variables of SSB, ASB, and natural juice for per 100 g/day increment consumption.

Abbreviation: IBS, irritable bowel syndrome.

**FIGURE 1:**
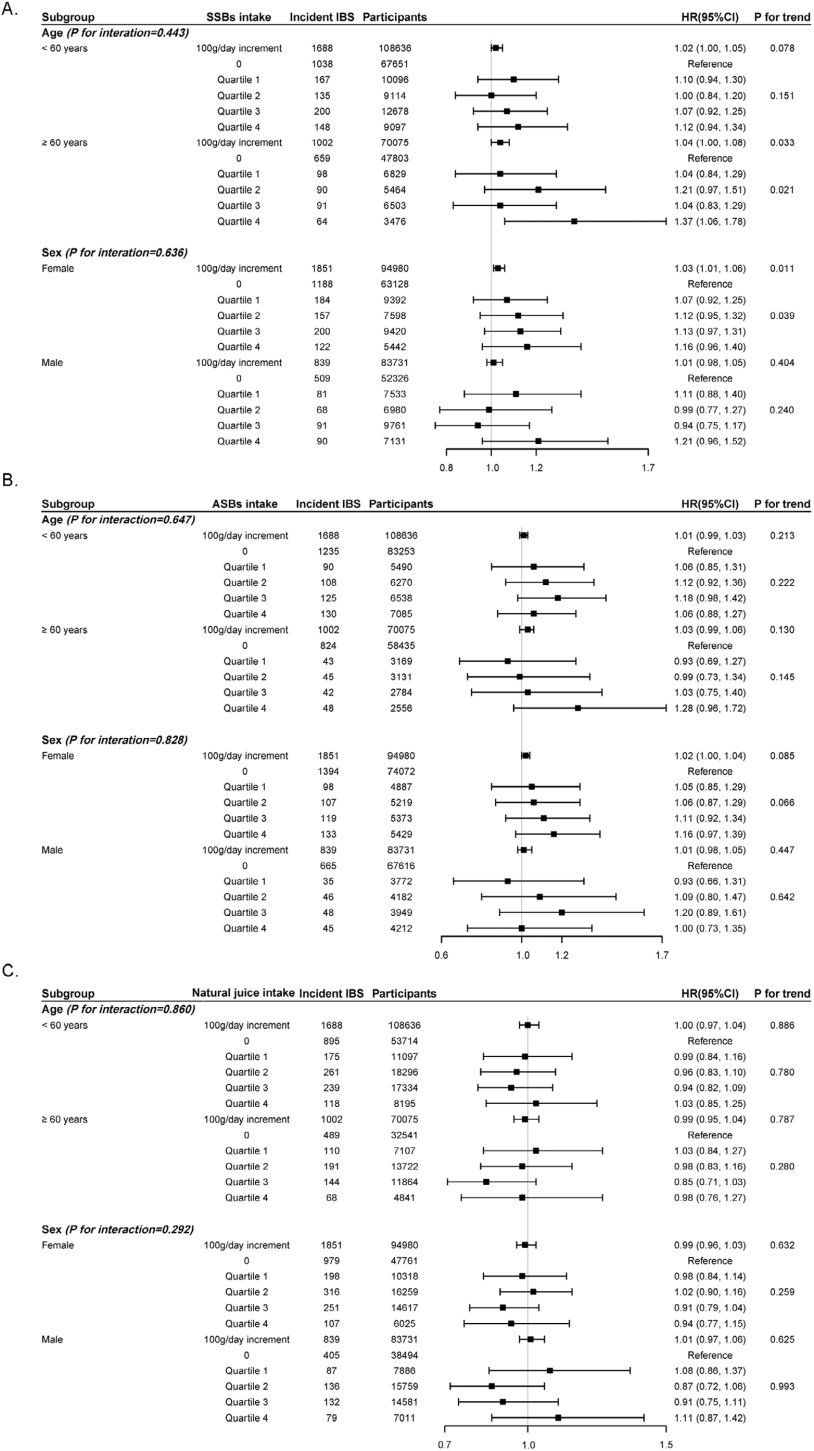
Association of sugar‐sweetened beverages, artificially sweetened beverages, and fruit juice consumption with incident IBS by age and sex. (A) Sugar‐sweetened beverages; (B) artificially sweetened beverages; (C) natural juice. All HRs were calculated by adjusting the following covariates: age, sex, Townsend deprivation index, education level, ethnicity, smoking status, alcohol drinking, IPAQ (International Physical Activity Questionnaire), total energy intake, type 2 diabetes, depression, and anxiety. *p* for trend was calculated by using median value (82.5, 130, 250, and 500 g/day) of each sugar‐sweetened beverages quartile, median value (82.5, 165, 330 and 660 g/day) of each artificially sweetened beverages quartile, and median value (62.5, 125, 250 and 417 g/day) of each natural juice quartile. Test for trend of per 100 g/day increment was performed by considering intake a continuous variable. CI, confidence interval; HR, hazard ratio; IBS, irritable bowel syndrome.

In subgroup analysis, higher IBS risk associated with SSB consumption (both per 100 g/day increment and quartiles) was generally observed across those with age ≥ 60 years, female, BMI > 25 kg/m^2^, never smoking, current alcohol drinking, and unhealthy diet pattern subgroups (Figure 1A, Tables [Link], [Link]). No significant interaction across these subgroups was observed (all *p*_interaction_ > 0.05).

### 3.3 ASB Intake and Risk of Incident IBS

The 11‐year cumulative incidence of IBS was 1.8% (95% CI: 1.5%–2.1%) in the highest quartile group of ASB intake versus 1.5% (95% CI: 1.4%–1.5%) in no ASB drinkers. No significant association with IBS occurrence was detected in those who consumed the highest quartile of ASB compared with no ASB intake (HR_Q4 VS no intake_ = 1.12, 95% CI: 0.95–1.31, *p*_trend_ = 0.062) after multivariable adjustment (Table 2, Table S8).

Similarly, consistent findings were observed in subgroup analyses by age, sex, BMI > 25 kg/m^2^, smoking, alcohol drinking, and diet pattern (Figure 1B, Tables [Link], [Link]). No significant modification effect was detected across those subgroups (all *p*_interaction_ > 0.05) except for BMI subgroups (*p*_interaction_ = 0.002), with higher IBS risk in those with BMI ≤ 25 kg/m^2^ (HR_Q4 VS no intake_ = 1.72, 95% CI: 1.30–2.27).

### 3.4 Natural Juice Intake and Risk of Incident IBS

Regarding natural juice intake, the 11‐year cumulative incidence of IBS was 1.4% (95% CI: 1.2%–1.6%) in the highest quartile group versus 1.6% (95% CI: 1.5%–1.7%) in no drinkers. Compared with no natural juice intake, no significant association with IBS occurrence was detected in those who consumed the highest quartile (HR_Q4 VS no intake_ = 1.01, 95% CI: 0.87–1.18, *p*_trend_ = 0.363) (Table 2, Table S9).

Consistent results were demonstrated in subgroup analyses by age, sex, BMI, smoking, alcohol drinking, and diet pattern (Figure 1C, Tables [Link], [Link]). No significant interaction was detected across those subgroups (all *p*_interaction_ > 0.05).

### 3.5 Total Sugar, Added Sugar, and Naturally Occurring Sugar Intake With Risk of Incident IBS

**FIGURE 2:**
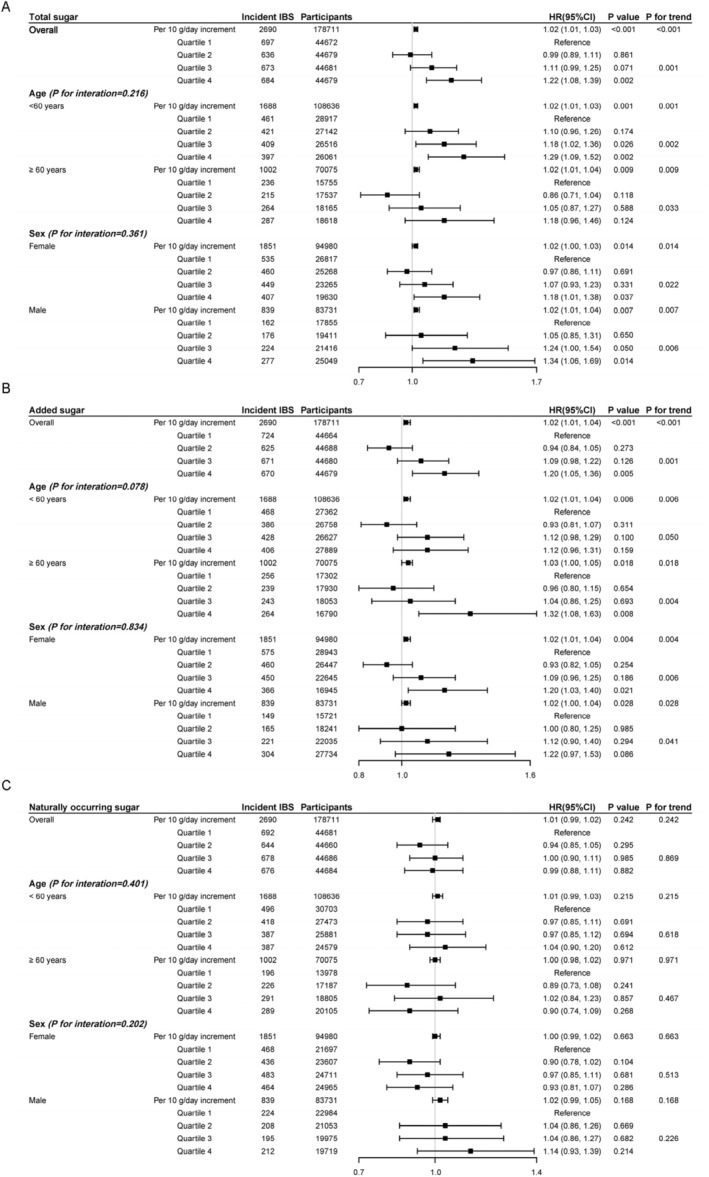
Association between total sugar, added sugar, and naturally occurring sugar intake and incident IBS. (A) total sugar; (B) added sugar; (C) naturally occurring sugar. All HRs were adjusted by age, sex, BMI, Townsend deprivation index, education level, ethnicity, smoking status and alcohol drinking, IPAQ (International Physical Activity Questionnaire), total energy intake, type 2 diabetes, depression, and anxiety; *p* for trend was calculated by using median value of each total sugar quartile (74.2, 106, 133, and 177 g/day), each added sugar quartile (28.2, 49.4, 69.6, and 104 g/day), and each naturally occurring sugar quartile (30.8, 48.9, 65.0, and 90.1 g/day), and considering intake a continuous variable for per 10 g/day increment of total sugar, added sugar, and naturally occurring sugar intake. CI, confidence interval; HR, hazard ratio; IBS, irritable bowel syndrome.

Participants with the highest quartile of total sugar intake compared with the lowest had a higher risk of incident IBS by 22% (HR = 1.22, 95% CI: 1.08–1.39, *p*_trend_ = 0.001, Figure 2A). Similarly, a 2% increased risk of IBS was observed accompanied by a per 10 g/day increment of total sugar intake (HR = 1.02, 95% CI: 1.01–1.03, *p*_trend_ < 0.001). Considering the intake of sugar forms, the increased risk of IBS was detected in added sugar (HR_Q4 VS Q1_ = 1.20, 95% CI: 1.05–1.36, *p*_trend_ = 0.001, Figure 2B), instead of naturally occurring sugar (HR_Q4 VS Q1_ = 0.99, 95% CI: 0.88–1.11, *p*_trend_ = 0.869, Figure 2C). Consistently, a 2% higher risk of IBS was observed with a per 10 g/day increment of added sugar intake (HR = 1.02, 95% CI: 1.01–1.04, *p*_trend_ < 0.001, Figure 2B), whereas a null association was detected with a per 10 g/day increment of naturally occurring sugar intake (Figure 2C).

Meanwhile, the evidently elevated risk of incident IBS with increasing levels of total sugar and added sugar consumption persisted across all age and sex subgroups with significant dose–response relationships (all *p*_trend_ < 0.05), while a null association with naturally occurring sugars was observed among those subgroups (all *p* values > 0.05, Figure 2A–C).

### 3.6 Sensitivity Analysis

Results of sensitivity analysis for SSB, ASB, and natural juice intake, either in quartiles or per 100 g/day increment, were all consistent with principal findings when excluding incident IBS cases within 1 or 2 years after the 24‐h dietary recall questionnaire, mutually adjusting SSB and natural juice, without adjusting total energy intake, performing competing risk models, additionally adjusting dietary pattern, excluding participants with different high and low cutoff points for energy intake, or using quintiles of beverage consumption (Table S10).

## 4 Discussion

In this large‐scale prospective cohort study, we found higher SSB intake was associated with a greater risk of IBS. By contrast, no association was detected between consumption of ASB as well as natural juice and the risk of IBS. More specifically, consumption of added sugar, rather than naturally occurring sugar, was associated with an increased risk of IBS. Every 10 g/day increment in added sugar consumption was associated with an increased incidence of IBS by 2%.

According to the national survey data from the USA, the average intake of SSBs remained high since 2003, contributing 145 kcal per day and corresponding to 6.5%–10% of daily calories (Marriott et al. 2019; Malek et al. 2018). As an important component of the diet, SSBs have been found to have a variety of adverse effects on human health (Malik and Hu 2022; Huang et al. 2023). However, the association of SSB consumption and IBS risk has long been controversial. Recent studies conducted in Norway and India revealed greater odds of IBS in individuals with higher consumption of carbonated beverages or aerated soft drinks (Ligaarden et al. 2012; Ghoshal and Singh 2017). By contrast, a null association with IBS or functional bowel disorders was observed in the French Nutrinet‐Santé study and other surveys in China (Xu et al. 2021; Guo et al. 2015; Torres et al. 2018). Nonetheless, all these studies were cross‐sectional with prevalent IBS cases rather than a cohort design with incident IBS, thereby leading to potential bias of reverse causation. Moreover, most studies had neither a clear definition of carbonated/soft drinks (i.e., combining SSB and ASB together) nor a thorough adjustment of important confounders, resulting in masking important differences and residual confounding. All of these may explain the discrepancies between previous studies and ours. Taken together, our study addressed these knowledge gaps and provided unique epidemiological evidence on the harmful effect of SSB as well as added sugar intake for IBS development.

Several potential biological mechanisms might explain the increased IBS risk associated with higher SSB consumption. Firstly, SSB, mainly sweetened by high‐fructose corn syrup (HFCS, containing 42%–55% fructose and 45%–58% glucose) in the USA, sucrose (50% fructose and 50% glucose) in Europe, or fruit juice concentrates (mainly fructose), is the major source of FODMAP in the diet. Excessive dietary intake of these monosaccharides and disaccharides can overwhelm small intestinal absorptive capacity, leading to malabsorption. Herein, unabsorbed sugars rapidly ferment in the bacteria‐rich colon and cause gas production, bloating, and diarrhea, which may contribute to IBS pathogenesis (Lacy et al. 2021; McKenzie et al. 2016; Singh and Nee 2021). Secondly, high intake of fructose in SSBs has been demonstrated to enhance the process of hepatic de novo lipogenesis, which may subsequently promote ectopic fat deposition in the liver (Stanhope and Havel 2008). A recent umbrella review showed a 39% greater risk of non‐alcoholic fatty liver disease (NAFLD) associated with high SSB intake (Huang et al. 2023). Thus, the excess risk of IBS associated with SSB may be mediated by an increased risk of NAFLD based on prior epidemiological evidence (Wu et al. 2022). Thirdly, SSB may promote the IBS pathogenic process through modulating gut microbiota (i.e., decreased diversity and alternations of amount), increasing mucosal inflammation, and disturbing the intestinal barrier (Malik and Hu 2022; Khan et al. 2020; O'Connor et al. 2018). Additionally, as IBS has been considered a disorder of gut‐brain interaction, SSB may increase IBS risk by modulating the gut‐brain axis via several neuronal pathways, including changes in the dopaminergic system and vagal neurons (Tan et al. 2020). However, the exact underlying mechanisms remain to be fully elucidated.

In line with other studies, our study did not observe any harmful or beneficial effect of natural juice on IBS risk, although natural juice also mainly contained fructose (Ligaarden et al. 2012; Xu et al. 2021; Guo et al. 2015; Torres et al. 2018). The large Nutrinet‐Santé study reported a null association between 100% vegetable/fruit juice consumption and prevalent IBS (Torres et al. 2018). This finding may be explained by the beneficial effects of vitamins and other bioactive substances in fruit juices, which might offset the harmful consequences of rapidly absorbed monosaccharides and disaccharides (Malik and Hu 2022). However, owing to data availability and a variety of complex bioactive substances in natural juice, we could not explicitly test the direct associations between these correlated factors and incident IBS risk and verify the hypothesized mechanism. Another possible explanation may be the different roles of different sugar forms in IBS pathogenesis, since the main component of SSB and natural juice is separately added sugar and naturally occurring sugars (Gonzalez 2024; Gillespie et al. 2023). Interestingly, our findings further confirmed the higher IBS risk associated with added sugars but not naturally occurring sugars, suggesting a potential role of sugar forms rather than simply the amount. Nevertheless, the exact mechanisms involved in such roles of intrinsic and extrinsic sugars remain to be investigated in detail.

Although ASB is considered a healthier alternative owing to its few calories and no sugar, little is known regarding its effect on the risk of IBS development. Interestingly, we did not detect any increased risk of IBS occurrence associated with ASB consumption except for individuals with BMI ≤ 25 kg/m^2^. However, evidence from cellular and animal studies demonstrated that artificial sweeteners may decrease gut mobility and delay intestinal transit by stimulating gut hormones release (i.e., glucagon‐like peptide‐1, gastric inhibitory polypeptide, Peptide YY, and cholecystokinin) and serotonin (Spencer et al. 2016; Ogawa et al. 2011). Meanwhile, accumulating evidence from animal studies indicated the effect of artificial sweeteners on alterations of gut microflora, with a significant decrease of beneficial intestinal bacteria (i.e., Lactobacillus and Bifidobacterium) (Spencer et al. 2016; Suez et al. 2014). Hence, all these may adversely affect intestinal permeability and visceral sensation, which, in turn, may potentially increase the risk of incident IBS and be a potential explanation for the higher IBS risk in those with BMI ≤ 25 kg/m^2^. Nonetheless, to date, a remarkable paucity of literature addresses this issue and whether ASB could be a safe substitute remains to be further validated. Besides, different artificial sweeteners are approved to be used in different countries. Currently, the Food and Drug Administration (FDA) allows 8 kinds of sweeteners to be used in the US, including Acesulfame potassium, Advantame, Aspartame, Neotame, Saccharin, Sucralose, Luo Han Guo, and Purified stevia leaf extracts. By contrast, the UK has more sweetener options than the US. In addition to the first 6 sweeteners in the US, other 15 kinds of sweeteners are allowed to be used according to the European Food Safety Agency (EFSA). Ongoing re‐evaluation of different kinds of sweeteners should also be necessary.

To the best of our knowledge, our findings for the first time highlighted the long‐term risk of IBS associated with SSB, ASB, and natural juice consumption, based on the well‐designed prospective cohort design. Moreover, we thoroughly examined the relationship with different sugar forms by adjusting for numerous potential confounders, confirming a greater IBS risk associated with SSB and added sugar intake. Additionally, various sensitivity analyses, by accounting for reverse causation and information bias, were conducted, verifying the robustness of results.

However, several potential limitations should be considered. Firstly, SSB, ASB, and natural juice consumption were self‐reported through a validated 24 h diet recall questionnaire, which was susceptible to measurement error and recall bias. However, we calculated the average value of dietary intake from multiple cycles after excluding participants with implausible energy intake, reducing the risk of measurement error. Secondly, SSB, ASB, and natural juice consumption were only measured once at baseline, instead of through repeated measurements. Hence, the IBS risk associated with changes in those beverages consumption could not be assessed. Thirdly, owing to unavailable IBS subtype data (i.e., IBS‐Diarrhea, IBS‐Constipation, IBS‐Mixed), we only investigated the associated risk of IBS as a whole, rather than distinguishing among various IBS subtypes, which may limit the applicability of the findings to specific patient populations. Future research should explore these subtypes individually to provide a more nuanced understanding of IBS. Fourthly, incident IBS cases ascertained via only ICD‐10 code may be underestimated in this study, since some patients may not seek medical consultation. Thus, this may lead to misclassification bias. Subsequent studies should incorporate more rigorous diagnostic criteria (i.e., Rome IV criteria) to enhance the robustness of the findings. Fifthly, other lifestyle factors or dietary habits may vary during the long‐term follow‐up, which may confound the association. Sixthly, although we have carefully controlled for various potential confounders, residual confounding may still exist owing to the observational design. Seventhly, despite the relatively large‐scale population, limited statistical power owing to insufficient subgroup sample size could not be avoided, leading to false‐negative results. Eighthly, owing to the unavailable data, we could not compare the risk of incident IBS among those who drink different types of 100% fruit juice (i.e., between those who drink mostly 100% orange juice and those who drink 100% grape juice) and different types of artificial sweeteners. Ninthly, the exact biological pathways or channels for the increased IBS risk of SSBs rather than ASBs and natural juice is still unclear and remain to be investigated in detail. Finally, our study was conducted among predominantly White Europeans of relatively older age, which may limit the generalizability of our findings to other ethnic populations and different age periods.

## 5 Conclusions

In summary, this large population‐based prospective cohort demonstrated that higher intake of sugar‐sweetened beverages was associated with a greater risk of incident IBS, with a significant dose–response relationship. Artificially sweetened beverages and natural juice intake appeared to have no association with the incidence of IBS. Meanwhile, higher consumption of added sugar, instead of naturally occurring sugar, was associated with a higher risk of IBS. Although causality cannot be inferred due to the observational design, these findings highlight the importance of limiting SSB consumption in diets to reduce the modifiable burden of IBS. Given the increasing trend in both SSB consumption and disease burden, future health‐related policies should incorporate these findings to alleviate the adverse impact of SSB on IBS development. Further studies are required to validate these findings in diverse ethnic populations and clarify underlying biological mechanisms.

## Author Contributions

S Wu and S Zhu designed the study. S Wu drafted the manuscript. S Wu analyzed the data. Z Yang and Q Zhang revised the manuscript. S Wu, Z Yang, S Liu, Q Zhang, S Zhu, and S Zhang interpreted the results, incorporated comments from the co‐authors, and finalized the manuscript. All authors approved the final version of the paper.

## Ethics Statement

The UKB study was approved by the North West Multicenter Research Ethical Committee(21/NW/0157), and all participants or their proxy respondents provided written informed consent.

## Conflicts of Interest

The authors declare no conflicts of interest.

## Supporting information


Table S1.

Table S2.

Table S3.

Table S4.

Table S5.

Table S6.

Table S7.

Table S8.

Table S9.

Table S10.

Table S11.

## Data Availability

All data relevant to the study were obtained using the UK Biobank Resource under application number [74444]. No additional data are available.

## References

[fsn370094-bib-0001] Frändemark, Å. , H. Törnblom , S. Jakobsson , and M. Simrén . 2018. “Work Productivity and Activity Impairment in Irritable Bowel Syndrome (IBS): A Multifaceted Problem.” American Journal of Gastroenterology 113: 1540–1549. 10.1038/s41395-018-0262-x. 30254230

[fsn370094-bib-0002] Ghoshal, U. C. , and R. Singh . 2017. “Frequency and Risk Factors of Functional Gastro‐Intestinal Disorders in a Rural Indian Population.” Journal of Gastroenterology and Hepatology 32: 378–387. 10.1111/jgh.13465. 27262283

[fsn370094-bib-0003] Gillespie, K. M. , E. Kemps , M. J. White , and S. E. Bartlett . 2023. “The Impact of Free Sugar on Human Health‐A Narrative Review.” Nutrients 15, no. 4: 889. 10.3390/nu15040889. 36839247 PMC9966020

[fsn370094-bib-0004] Gonzalez, J. T. 2024. “Are all Sugars Equal? Role of the Food Source in Physiological Responses to Sugars With an Emphasis on Fruit and Fruit Juice.” European Journal of Nutrition 63: 1435–1451. 10.1007/s00394-024-03365-3. 38492022 PMC11329689

[fsn370094-bib-0005] Guo, Y. B. , K. M. Zhuang , L. Kuang , Q. Zhan , X. F. Wang , and S. D. Liu . 2015. “Association Between Diet and Lifestyle Habits and Irritable Bowel Syndrome: A Case‐Control Study.” Gut Liver 9: 649–656. 10.5009/gnl13437. 25266811 PMC4562783

[fsn370094-bib-0006] Hess, K. R. 1995. “Graphical Methods for Assessing Violations of the Proportional Hazards Assumption in Cox Regression.” Statistics in Medicine 14: 1707–1723. 10.1002/sim.4780141510. 7481205

[fsn370094-bib-0007] Huang, Y. , Z. Chen , B. Chen , et al. 2023. “Dietary Sugar Consumption and Health: Umbrella Review.” BMJ 381: e071609. 10.1136/bmj-2022-071609. 37019448 PMC10074550

[fsn370094-bib-0008] Khan, S. , S. Waliullah , V. Godfrey , et al. 2020. “Dietary Simple Sugars Alter Microbial Ecology in the Gut and Promote Colitis in Mice.” Science Translational Medicine 12: eaay6218. 10.1126/scitranslmed.aay6218. 33115951

[fsn370094-bib-0009] Lacy, B. E. , M. Pimentel , D. M. Brenner , D. C. William , A. K. Laurie , and D. L. Millie . 2021. “ACG Clinical Guideline: Management of Irritable Bowel Syndrome.” American Journal of Gastroenterology 116: 17–44. 10.14309/ajg.0000000000001036. 33315591

[fsn370094-bib-0010] Ligaarden, S. C. , S. Lydersen , and P. G. Farup . 2012. “Diet in Subjects With Irritable Bowel Syndrome: A Cross‐Sectional Study in the General Population.” BMC Gastroenterology 12: 61. 10.1186/1471-230X-12-61. 22676475 PMC3674839

[fsn370094-bib-0011] Malek, A. M. , K. J. Hunt , D. M. DellaValle , D. Greenberg , J. V. S. Peter , and B. P. Marriott . 2018. “Reported Consumption of Low‐Calorie Sweetener in Foods, Beverages, and Food and Beverage Additions by US Adults: NHANES 2007–2012.” Current Developments in Nutrition 2: nzy054. 10.1093/cdn/nzy054. 30283913 PMC6163106

[fsn370094-bib-0012] Malik, V. S. , and F. B. Hu . 2022. “The Role of Sugar‐Sweetened Beverages in the Global Epidemics of Obesity and Chronic Diseases.” Nature Reviews. Endocrinology 18: 205–218. 10.1038/s41574-021-00627-6. PMC8778490 35064240

[fsn370094-bib-0013] Marriott, B. P. , K. J. Hunt , A. M. Malek , and J. C. Newman . 2019. “Trends in Intake of Energy and Total Sugar From Sugar‐Sweetened Beverages in the United States Among Children and Adults, NHANES 2003–2016.” Nutrients 11, no. 9: 2004. 10.3390/nu11092004. 31450689 PMC6770750

[fsn370094-bib-0014] McKenzie, Y. A. , R. K. Bowyer , H. Leach , et al. 2016. “British Dietetic Association Systematic Review and Evidence‐Based Practice Guidelines for the Dietary Management of Irritable Bowel Syndrome in Adults (2016 Update).” Journal of Human Nutrition and Dietetics 29: 549–575. 10.1111/jhn.12385. 27272325

[fsn370094-bib-0015] Mozaffarian, D. 2016. “Dietary and Policy Priorities for Cardiovascular Disease, Diabetes, and Obesity: A Comprehensive Review.” Circulation 133: 187–225. 10.1161/CIRCULATIONAHA.115.018585. 26746178 PMC4814348

[fsn370094-bib-0016] O'Connor, L. , F. Imamura , S. Brage , S. J. Griffin , N. J. Wareham , and N. G. Forouhi . 2018. “Intakes and Sources of Dietary Sugars and Their Association With Metabolic and Inflammatory Markers.” Clinical Nutrition 37: 1313–1322. 10.1016/j.clnu.2017.05.030. 28711418 PMC5999353

[fsn370094-bib-0017] Ogawa, E. , M. Hosokawa , N. Harada , et al. 2011. “The Effect of Gastric Inhibitory Polypeptide on Intestinal Glucose Absorption and Intestinal Motility in Mice.” Biochemical and Biophysical Research Communications 404: 115–120. 10.1016/j.bbrc.2010.11.077. 21095180

[fsn370094-bib-0018] Perez‐Cornago, A. , Z. Pollard , H. Young , et al. 2021. “Description of the Updated Nutrition Calculation of the Oxford WebQ Questionnaire and Comparison With the Previous Version Among 207,144 Participants in UK Biobank.” European Journal of Nutrition 60: 4019–4030. 10.1007/s00394-021-02558-4. 33956230 PMC8437868

[fsn370094-bib-0019] Piernas, C. , A. Perez‐Cornago , M. Gao , et al. 2021. “Describing a New Food Group Classification System for UK Biobank: Analysis of Food Groups and Sources of Macro‐ and Micronutrients in 208,200 Participants.” European Journal of Nutrition 60: 2879–2890. 10.1007/s00394-021-02535-x. 33768317 PMC8275520

[fsn370094-bib-0020] Singh, P. , and J. Nee . 2021. “Role of Diet in Diarrhea‐Predominant Irritable Bowel Syndrome.” Journal of Clinical Gastroenterology 55: 25–29. 10.1097/MCG.0000000000001445. 33060435

[fsn370094-bib-0021] Spencer, M. , A. Gupta , L. V. Dam , C. Shannon , S. Menees , and W. D. Chey . 2016. “Artificial Sweeteners: A Systematic Review and Primer for Gastroenterologists.” Journal of Neurogastroenterology and Motility 22: 168–180. 10.5056/jnm15206. 26932837 PMC4819855

[fsn370094-bib-0022] Sperber, A. D. , S. I. Bangdiwala , D. A. Drossman , et al. 2021. “Worldwide Prevalence and Burden of Functional Gastrointestinal Disorders, Results of Rome Foundation Global Study.” Gastroenterology 160: 99–114. 10.1053/j.gastro.2020.04.014. 32294476

[fsn370094-bib-0023] Stanhope, K. L. , and P. J. Havel . 2008. “Endocrine and Metabolic Effects of Consuming Beverages Sweetened With Fructose, Glucose, Sucrose, or High‐Fructose Corn Syrup.” American Journal of Clinical Nutrition 88: 1733S–1737S. 10.3945/ajcn.2008.25825D. 19064538 PMC3037017

[fsn370094-bib-0024] Sudlow, C. , J. Gallacher , N. Allen , et al. 2015. “UK Biobank: An Open Access Resource for Identifying the Causes of a Wide Range of Complex Diseases of Middle and Old Age.” PLoS Medicine 12: e1001779. 10.1371/journal.pmed.1001779. 25826379 PMC4380465

[fsn370094-bib-0025] Suez, J. , T. Korem , D. Zeevi , et al. 2014. “Artificial Sweeteners Induce Glucose Intolerance by Altering the Gut Microbiota.” Nature 514: 181–186. 10.1038/nature13793. 25231862

[fsn370094-bib-0026] Tan, H. E. , A. C. Sisti , H. Jin , et al. 2020. “The Gut‐Brain Axis Mediates Sugar Preference.” Nature 580: 511–516. 10.1038/s41586-020-2199-7. 32322067 PMC7185044

[fsn370094-bib-0027] Torres, M. J. , J. M. Sabate , M. Bouchoucha , C. Buscail , S. Hercberg , and C. Julia . 2018. “Food Consumption and Dietary Intakes in 36,448 Adults and Their Association With Irritable Bowel Syndrome: Nutrinet‐Santé Study.” Therapeutic Advances in Gastroenterology 11: 1756283X17746625. 10.1177/1756283X17746625. PMC5788087 29399039

[fsn370094-bib-0028] Wu, S. , C. Yuan , Z. Yang , et al. 2022. “Non‐Alcoholic Fatty Liver Is Associated With Increased Risk of Irritable Bowel Syndrome: A Prospective Cohort Study.” BMC Medicine 20: 262. 10.1186/s12916-022-02460-8. 35989356 PMC9394037

[fsn370094-bib-0029] Xu, Z. Y. , C. Chen , Z. Ouyang , et al. 2021. “Beverages Intake and Functional Bowel Disorders: A Cross‐Sectional Study in First‐Year Undergraduates.” Journal of Digestive Diseases 22: 630–636. 10.1111/1751-2980.13059. 34623731

