# Supplementary material for: Sugar‐Sweetened Beverages, Artificially Sweetened Beverages and Sugar Forms With Long‐Term Risk of Irritable Bowel Syndrome: A Large‐Scale Prospective Cohort Study
Source: Food Sci Nutr. 2025 Mar 19;13(3):e70094. doi: 10.1002/fsn3.70094 (PMC11922681; doi:10.1002/fsn3.70094)
Supplement: Supplementary file 1 — Table S1. [file FSN3-13-e70094-s001.docx]

**Table S1. Baseline characteristics according to baseline artificially sweetened beverages consumption in the cohort.**

| **Characteristic** | **Total**  **(N=178711)** | **0**  **(N=142228)** | **Quartile 1**  **(N=8696)** | **Quartile 2**  **(N=9432)** | **Quartile 3**  **(N=9368)** | **Quartile 4**  **(N=9678)** |
| --- | --- | --- | --- | --- | --- | --- |
| Age(years)^*^ | 55.81±7.96 | 56.23±7.89 | 55.36±7.81 | 54.66±7.99 | 53.84±8.02 | 53.03±8.04 |
| Sex |  |  |  |  |  |  |
| Male | 83731 (46.9) | 67616 (47.7) | 3772 (43.6) | 4182 (44.5) | 3949 (42.4) | 4212 (43.7) |
| Female | 94980 (53.1) | 740728 (52.3) | 4887 (56.4) | 5219 (55.5) | 5373 (57.6) | 5429 (56.3) |
| Nutrient and food intake |  |  |  |  |  |  |
| Total energy intake (KJ/day) ^*^ | 8648±2447 | 8664±2449 | 8596±2099 | 8581±2309 | 8520±2638 | 8646±2636 |
| Total daily intake (g/day) ^*#^ | 3221±799 | 3179±785 | 3182±687 | 3276±770 | 3365±841 | 3684±921 |
| Protein (g/day) ^*^ | 80.9±24.5 | 80.6±24.5 | 81.1±20.3 | 81.7±23.1 | 81.8±26.8 | 83.8±27.9 |
| Fat (g/day) ^*^ | 73.1±28.4 | 73.3±28.5 | 72.7±24.0 | 72.0±26.3 | 71.5±30.4 | 73.4±31.2 |
| Carbohydrate (g/day) ^*^ | 254.3±78.5 | 254.6±78.4 | 252.0 ±66.6 | 253.4±74.9 | 252.8±85.7 | 254.6±86.5 |
| Englyst fiber (g/day) ^*^ | 17.8±6.7 | 17.9±6.7 | 17.4±5.5 | 17.5±6.2 | 17.3±7.0 | 17.4±7.0 |
| Alcohol intake (g/day) ^*^ | 17.3±22.2 | 17.6±22.6 | 17.1±19.1 | 16.3±19.9 | 15.2±21.4 | 14.8±21.7 |
| Sugar-sweetened beverages (g/day) ^*^ | 90.0±181.5 | 85.0±179.5 | 97.1±138.2 | 108.4±162.1 | 104.9±191.9 | 125.1±237.4 |
| Artificially sweetened beverages (g/day) ^*^ | 72.4±198.3 | 0.0±0.0 | 88.7±23.9 | 188.5±34.3 | 330.9±6.9 | 758.8±317.9 |
| Natural juice (g/day) ^*^ | 105.7±144.5 | 109.2±146.7 | 100.5±116.1 | 95.6±135.5 | 89.1±139.1 | 84.7±145.5 |
| Total sugars intake (g/day) ^*^ | 124.8±48.7 | 125.1±48.6 | 123.0±41.2 | 123.8±46.4 | 122.5±52.5 | 123.8±55.0 |
| Ethnicity |  |  |  |  |  |  |
| Non-White | 8199 (4.6) | 6625 (4.7) | 310 (3.6) | 413 (4.4) | 477 (5.1) | 374 (3.9) |
| White | 169930 (95.1) | 134583 (95.0) | 8328 (96.2) | 8963 (95.3) | 8817 (94.6) | 9239 (95.8) |
| Unknown | 582 (0.3) | 480 (0.3) | 21 (0.2) | 25 (0.3) | 28 (0.3) | 28 (0.3) |
| Education level |  |  |  |  |  |  |
| Non-university | 100832 (56.4) | 78514 (55.4) | 4752 (54.9) | 5677 (60.4) | 5841 (62.7) | 6048 (62.7) |
| University | 77000 (43.1) | 62473 (44.1) | 3863 (44.6) | 3689 (39.2) | 3423 (36.7) | 3552 (36.8) |
| Unknown | 879 (0.5) | 701 (0.5) | 44 (0.5) | 35 (0.4) | 58 (0.6) | 41 (0.4) |
| Townsend deprivation index | | | | | | |
| Mean (SD) | -1.55 (2.88) | -1.55 (2.88) | -1.68 (2.82) | -1.66 (2.82) | -1.51 (2.94) | -1.35 (2.99) |
| Q1(≤ -3.71) | 44592 (25.0) | 35371 (25.0) | 2269 (26.2) | 2380 (25.3) | 2297 (24.6) | 2275 (23.6) |
| Q2(-3.71 - -2.30) | 44659 (25.0) | 35389 (25.0) | 2213 (25.6) | 2425 (25.8) | 2337 (25.1) | 2295 (23.8) |
| Q3(-2.30 - 0.11) | 44618 (25.0) | 35372 (25.0) | 2114 (24.4) | 2383 (25.3) | 2354 (25.3) | 2395 (24.8) |
| Q4 (>0.11) | 44614 (25.0) | 35401 (25.0) | 2046 (23.6) | 2198 (23.4) | 2320 (24.9) | 2649 (27.5) |
| Unknown | 228 (0.1) | 155 (0.1) | 17 (0.2) | 15 (0.2) | 14 (0.2) | 27 (0.3) |
| Smoking status |  |  |  |  |  |  |
| Never | 101498 (56.8) | 80801 (57.0) | 4895 (56.5) | 5305 (56.4) | 5272 (56.6) | 5225 (54.2) |
| Previous | 62545 (35.0) | 49216 (34.7) | 3164 (36.5) | 3363 (35.8) | 3297 (35.4) | 3505 (36.4) |
| Current | 14191 (7.9) | 11304 (8.0) | 567 (6.5) | 710 (7.6) | 734 (7.9) | 876 (9.1) |
| Unknown | 477 (0.3) | 367 (0.3) | 33 (0.4) | 23 (0.2) | 19 (0.2) | 35 (0.4) |
| Alcohol drinking |  |  |  |  |  |  |
| Never | 5741 (3.2) | 4471 (3.2) | 245 (2.8) | 294 (3.1) | 332 (3.6) | 399 (4.1) |
| Previous | 5221 (2.9) | 3921 (2.8) | 243 (2.8) | 242 (2.6) | 332 (3.6) | 483 (5.0) |
| Current | 167571 (93.8) | 133153 (94.0) | 8165 (94.3) | 8854 (94.2) | 8653 (92.8) | 8746 (90.7) |
| Unknown | 178 (0.1) | 143 (0.1) | 6 (0.1) | 11 (0.1) | 5 (0.1) | 13 (0.1) |
| IPAQ |  |  |  |  |  |  |
| Low | 27668 (15.5) | 21474 (15.2) | 1444 (16.7) | 1495 (15.9) | 1527 (16.4) | 1728 (17.9) |
| Moderate | 64193 (35.9) | 51085 (36.1) | 3209 (37.1) | 3429 (36.5) | 3269 (35.1) | 3201 (33.2) |
| High | 60110 (33.6) | 47712 (33.7) | 2838 (32.8) | 3195 (34.0) | 3148 (33.8) | 3217 (33.4) |
| Unknown | 26740 (15.0) | 21417 (15.1) | 1168 (13.5) | 1282 (13.6) | 1378 (14.8) | 1495 (15.5) |
| BMI |  |  |  |  |  |  |
| <18.5 kg/m^2^ | 915 (0.5) | 823 (0.6) | 25 (0.3) | 32 (0.3) | 17 (0.2) | 18 (0.2) |
| 18.5-24.9 kg/m^2^ | 63104 (35.3) | 53955 (38.1) | 2680 (31.0) | 2475 (26.3) | 2134 (22.9) | 1860 (19.3) |
| 25.0-29.9 kg/m^2^ | 75429 (42.2) | 59684 (42.1) | 3818 (44.1) | 4125 (43.9) | 3987 (42.8) | 3815 (39.6) |
| ≥30 kg/m^2^ | 38765 (21.7) | 26825 (18.9) | 2113 (24.4) | 2753 (29.3) | 3159 (33.9) | 3915 (40.6) |
| Unknown | 498 (0.3) | 401 (0.3) | 23 (0.3) | 16 (0.2) | 25 (0.3) | 33 (0.3) |
| Type 2 Diabetes | 3498 (2.0) | 2241 (1.6) | 187 (2.2) | 283 (3.0) | 337 (3.6) | 450 (4.7) |
| Depression | 12297 (6.9) | 9310 (6.6) | 585 (6.8) | 655 (7.0) | 763 (8.2) | 984 (10.2) |
| Anxiety | 5581 (3.1) | 4219 (3.0) | 297 (3.4) | 329 (3.5) | 315 (3.4) | 421 (4.4) |

Note: Numbers are n (%) unless otherwise stated. *: displayed as mean±standard deviation. IPAQ: International Physical Activity Questionnaire; #: Estimated intake of total food weight based on food and beverage consumption yesterday; BMI: body mass index.
