# Supplementary material for: Sugar‐Sweetened Beverages, Artificially Sweetened Beverages and Sugar Forms With Long‐Term Risk of Irritable Bowel Syndrome: A Large‐Scale Prospective Cohort Study
Source: Food Sci Nutr. 2025 Mar 19;13(3):e70094. doi: 10.1002/fsn3.70094 (PMC11922681; doi:10.1002/fsn3.70094)
Supplement: Supplementary file 2 — Table S2. [file FSN3-13-e70094-s007.docx]

**Table S2. Baseline characteristics according to baseline natural juice consumption in the cohort.**

| **Characteristic** | **Total**  **(N=178711)** | **0**  **(N=86255)** | **Quartile 1**  **(N=18204)** | **Quartile 2**  **(N=32018)** | **Quartile 3**  **(N=29198)** | **Quartile 4**  **(N=13036)** |
| --- | --- | --- | --- | --- | --- | --- |
| Age(years)^*^ | 55.81±7.96 | 55.52±8.00 | 55.88±7.82 | 56.52±7.84 | 55.98±7.99 | 55.41±8.02 |
| Sex |  |  |  |  |  |  |
| Male | 83731 (46.9) | 38494 (44.6) | 7886 (43.3) | 15759 (49.2) | 14581 (49.9) | 7011 (53.8) |
| Female | 94980 (53.1) | 47761 (55.4) | 10318 (56.7) | 16259 (50.8) | 14617 (50.1) | 6025 (46.2) |
| Nutrient and food intake |  |  |  |  |  |  |
| Total energy intake (KJ/day) ^*^ | 8648±2447 | 8356±2519 | 8575±2059 | 8761±2279 | 9028±2426 | 9552±2557 |
| Total daily intake (g/day) ^*#^ | 3221±799 | 3127±813 | 3140±677 | 3224±732 | 3379±783 | 3597±894 |
| Protein (g/day) ^*^ | 80.9±24.5 | 79.7±25.8 | 80.4±20.1 | 80.9±22.4 | 82.7±24.5 | 85.6±26.2 |
| Fat (g/day) ^*^ | 73.1±28.4 | 71.7±29.4 | 74.2±24.2 | 73.8±26.8 | 74.3±28.8 | 76.9±29.7 |
| Carbohydrate (g/day) ^*^ | 254.3±78.5 | 241.5±80.1 | 247.9 ±64.9 | 259.0±71.7 | 271.7±76.0 | 297.9±83.3 |
| Englyst fiber (g/day) ^*^ | 17.8±6.7 | 17.2±6.9 | 17.7±5.6 | 18.1±6.2 | 18.7±6.6 | 19.7±7.2 |
| Alcohol intake (g/day) ^*^ | 17.3±22.2 | 16.8±23.0 | 17.2±19.1 | 17.7±21.0 | 18.2±22.8 | 16.8±21.8 |
| Sugar-sweetened beverages (g/day) ^*^ | 90.0±181.5 | 90.8±193.3 | 86.2±140.0 | 85.2±159.2 | 87.9±176.1 | 106.2±211.9 |
| Artificially sweetened beverages (g/day) ^*^ | 72.4±198.3 | 85.2±223.6 | 67.0±163.3 | 60.1±167.7 | 58.1±173.8 | 57.9±179.6 |
| Natural juice (g/day) ^*^ | 105.7±144.5 | 0.0±0.0 | 64.7±19.5 | 141.0±24.2 | 245.6±12.4 | 461.8±184.7 |
| Total sugars intake (g/day) ^*^ | 124.8±48.7 | 113.5±47.9 | 119.1±38.6 | 128.2±42.6 | 139.9±46.1 | 164.4±55.4 |
| Ethnicity |  |  |  |  |  |  |
| Non-White | 8199 (4.6) | 4321 (5.0) | 677 (3.7) | 1163 (3.6) | 1135 (3.9) | 903 (6.9) |
| White | 169930 (95.1) | 81650 (94.7) | 17475 (96.0) | 30776 (96.1) | 27958 (95.8) | 12071 (92.6) |
| Unknown | 582 (0.3) | 284 (0.3) | 52 (0.3) | 79 (0.2) | 105 (0.4) | 62 (0.5) |
| Education level |  |  |  |  |  |  |
| Non-university | 100832 (56.4) | 53783 (62.4) | 9465 (52.0) | 16779 (52.4) | 14921 (51.1) | 5884 (45.1) |
| University | 77000 (43.1) | 31934 (37.0) | 8682 (47.7) | 15113 (47.2) | 14181 (48.6) | 7090 (54.4) |
| Unknown | 879 (0.5) | 538 (0.6) | 57 (0.3) | 126 (0.4) | 96 (0.3) | 62 (0.5) |
| Townsend deprivation index | | | | | | |
| Mean (SD) | -1.55 (2.88) | -1.41 (2.94) | -1.62 (2.82) | -1.78 (2.76) | -1.78 (2.79) | -1.37 (3.02) |
| Q1(≤ -3.71) | 44592 (25.0) | 20266 (23.5) | 4622 (25.4) | 8634 (27.0) | 7958 (27.3) | 3112 (23.9) |
| Q2(-3.71 - -2.30) | 44659 (25.0) | 21092 (24.5) | 4509 (24.8) | 8423 (26.3) | 7497 (25.7) | 3138 (24.1) |
| Q3(-2.30 - 0.11) | 44618 (25.0) | 21789 (25.3) | 4609 (25.3) | 7877 (24.6) | 7158 (24.5) | 3185 (24.4) |
| Q4 (>0.11) | 44614 (25.0) | 22988 (26.7) | 4433 (24.4) | 7055 (22.0) | 6546 (22.4) | 3592 (27.6) |
| Unknown | 228 (0.1) | 120 (0.1) | 31 (0.2) | 29 (0.1) | 39 (0.1) | 9 (0.1) |
| Smoking status |  |  |  |  |  |  |
| Never | 101498 (56.8) | 46532 (53.9) | 10488 (57.6) | 18760 (58.6) | 17774 (60.9) | 7944 (60.9) |
| Previous | 62545 (35.0) | 31319 (36.3) | 6487 (35.6) | 11135 (34.8) | 9510 (32.6) | 4094 (31.4) |
| Current | 14191 (7.9) | 8146 (9.4) | 1198 (6.6) | 2053 (6.4) | 1834 (6.3) | 960 (7.4) |
| Unknown | 477 (0.3) | 258 (0.3) | 31 (0.2) | 70 (0.2) | 80 (0.3) | 38 (0.3) |
| Alcohol drinking |  |  |  |  |  |  |
| Never | 5741 (3.2) | 3014 (3.5) | 499 (2.7) | 839 (2.6) | 858 (2.9) | 531 (4.1) |
| Previous | 5221 (2.9) | 2933 (3.4) | 462 (2.5) | 693 (2.2) | 696 (2.4) | 437 (3.4) |
| Current | 167571 (93.8) | 80204 (93.0) | 17235 (94.7) | 30459 (95.1) | 27620 (94.6) | 12053 (92.5) |
| Unknown | 178 (0.1) | 104 (0.1) | 8 (0.0) | 27 (0.1) | 24 (0.1) | 15 (0.1) |
| IPAQ |  |  |  |  |  |  |
| Low | 27668 (15.5) | 13745 (15.9) | 2928 (16.1) | 4869 (15.2) | 4203 (14.4) | 1923 (14.8) |
| Moderate | 64193 (35.9) | 29817 (34.6) | 6839 (37.6) | 11885 (37.1) | 10899 (37.3) | 4753 (36.5) |
| High | 60110 (33.6) | 28821 (33.4) | 5988 (32.9) | 10722 (33.5) | 9884 (33.9) | 4695 (36.0) |
| Unknown | 26740 (15.0) | 13872 (16.1) | 2449 (13.5) | 4542 (14.2) | 4212 (14.4) | 1665 (12.8) |
| BMI |  |  |  |  |  |  |
| <18.5 kg/m^2^ | 915 (0.5) | 472 (0.5) | 95 (0.5) | 155 (0.5) | 132 (0.5) | 61 (0.5) |
| 18.5-24.9 kg/m^2^ | 63104 (35.3) | 28454 (33.0) | 6914 (38.0) | 11957 (37.3) | 10994 (37.7) | 4785 (36.7) |
| 25.0-29.9 kg/m^2^ | 75429 (42.2) | 36197 (42.0) | 7490 (41.1) | 13751 (42.9) | 12425 (42.6) | 5566 (42.7) |
| ≥30 kg/m^2^ | 38765 (21.7) | 20877 (24.2) | 3656 (20.1) | 6073 (19.0) | 5578 (19.1) | 2581 (19.8) |
| Unknown | 498 (0.3) | 255 (0.3) | 49 (0.3) | 82 (0.3) | 69 (0.2) | 43 (0.3) |
| Type 2 Diabetes | 3498 (2.0) | 2048 (2.4) | 307 (1.7) | 530 (1.7) | 429 (1.5) | 184 (1.4) |
| Depression | 12297 (6.9) | 6479 (7.5) | 1289 (7.0) | 1873 (5.8) | 1780 (6.1) | 876 (6.7) |
| Anxiety | 5581 (3.1) | 2932 (3.4) | 566 (3.1) | 872 (2.7) | 837 (2.9) | 374 (2.9) |

Note: Numbers are n (%) unless otherwise stated. *: displayed as mean±standard deviation. IPAQ: International Physical Activity Questionnaire; #: Estimated intake of total food weight based on food and beverage consumption yesterday; BMI: body mass index.
