# Supplementary material for: Sugar‐Sweetened Beverages, Artificially Sweetened Beverages and Sugar Forms With Long‐Term Risk of Irritable Bowel Syndrome: A Large‐Scale Prospective Cohort Study
Source: Food Sci Nutr. 2025 Mar 19;13(3):e70094. doi: 10.1002/fsn3.70094 (PMC11922681; doi:10.1002/fsn3.70094)
Supplement: Supplementary file 3 — Table S3. [file FSN3-13-e70094-s003.docx]

**Table S3.** **Results of IBS risk associated with baseline sugar-sweetened beverages consumption.**

| **Variables** | **Category** | **HR (95%CI)** | **P value** |
| --- | --- | --- | --- |
| SSB consumption | 0 | Reference |  |
|  | Quartile 1 | 1.08 (0.95, 1.23) | 0.250 |
|  | Quartile 2 | 1.08 (0.94, 1.24) | 0.303 |
|  | Quartile 3 | 1.06 (0.94, 1.20) | 0.360 |
|  | Quartile 4 | 1.19 (1.03, 1.37) | 0.021 |
| Age | Per year | 1.00 (0.99, 1.00) | 0.382 |
| Sex | Male | Reference |  |
|  | Female | 1.86 (1.70, 2.02) | <0.001 |
| BMI | Normal | Reference |  |
|  | Underweight | 1.02 (0.62, 1.68) | 0.929 |
|  | Overweight | 0.98 (0.90, 1.07) | 0.715 |
|  | Obesity | 0.99 (0.89, 1.10) | 0.873 |
| TDI | Quartile 1 | Reference |  |
|  | Quartile 2 | 0.98 (0.88, 1.10) | 0.770 |
|  | Quartile 3 | 1.01 (0.90, 1.12) | 0.923 |
|  | Quartile 4 | 1.05 (0.94, 1.17) | 0.356 |
| Education | Non-university | Reference |  |
|  | University | 0.77 (0.71, 0.84) | <0.001 |
| Ethnicity | Non-White | Reference |  |
|  | White | 1.18 (0.97, 1.44) | 0.100 |
| Alcohol drinking | Never | Reference |  |
|  | Previous | 1.76 (1.35, 2.28) | <0.001 |
|  | Current | 1.01 (0.82, 1.24) | 0.940 |
| Smoking | Never | Reference |  |
|  | Previous | 1.08 (1.00, 1.18) | 0.057 |
|  | Current | 1.05 (0.91, 1.21) | 0.512 |
| IPAQ | Low | Reference |  |
|  | Moderate | 0.83 (0.74, 0.93) | 0.001 |
|  | High | 0.80 (0.71, 0.90) | <0.001 |
| Type 2 Diabetes | No | Reference |  |
|  | Yes | 1.47 (1.16, 1.86) | 0.002 |
| Depression |  | Reference |  |
|  |  | 1.80 (1.61, 2.02) | <0.001 |
| Anxiety |  | Reference |  |
|  |  | 1.84 (1.58, 2.14) | <0.001 |
| Total energy intake | Per 1 kJ | 1.00 (1.00, 1.00) | 0.164 |

Note: All HRs were calculated by adjusting the following covariates in addition to SSB quartiles as exposure: age, sex, BMI, Townsend deprivation index, education level, ethnicity, smoking status, alcohol drinking, IPAQ (International Physical Activity Questionnaire), total energy intake, type 2 diabetes, depression and anxiety. IBS: irritable bowel syndrome; HR: hazard ratio; CI: confidence interval.
