# Supplementary material for: Sugar‐Sweetened Beverages, Artificially Sweetened Beverages and Sugar Forms With Long‐Term Risk of Irritable Bowel Syndrome: A Large‐Scale Prospective Cohort Study
Source: Food Sci Nutr. 2025 Mar 19;13(3):e70094. doi: 10.1002/fsn3.70094 (PMC11922681; doi:10.1002/fsn3.70094)
Supplement: Supplementary file 4 — Table S4. [file FSN3-13-e70094-s009.docx]

**Table S4.** **Risk of IBS associated with baseline sugar-sweetened beverages, artificially sweetened beverages and natural juice consumption stratified by BMI.**

| **SSBs/ASBs/Natural juice consumption** | **Sugar-sweetened beverages** | | | **Artificially sweetened beverages** | | | **Natural juice** | | |
| --- | --- | --- | --- | --- | --- | --- | --- | --- | --- |
|  | **No. of IBS/**  **participants** | **HR (95%CI)** | **P for trend** | **No. of IBS/**  **participants** | **HR (95%CI)** | **P for trend** | **No. of IBS/ participants** | **HR (95%CI)** | **P for trend** |
| **BMI≤24.9 kg/m^2^** | | | | | | | | | |
| 100g/day increment | 987/64019 | 1.01 (0.97-1.05) | 0.593^*^ | 987/64019 | 1.05 (1.01-1.08) | 0.007^*^ | 987/64019 | 1.02 (0.97-1.06) | 0.497^*^ |
| 0 | 628/42185 | Reference |  | 819/54778 | Reference |  | 469/28926 | Reference |  |
| Quartile 1 | 125/6700 | 1.25 (1.03-1.52) |  | 51/2705 | 1.22 (0.92-1.62) |  | 109/7009 | 0.98 (0.80-1.21) |  |
| Quartile 2 | 73/5088 | 0.97 (0.76-1.24) | 0.477 | 37/2507 | 0.97 (0.69-1.34) | 0.004 | 178/12112 | 0.97 (0.81-1.15) | 0.835 |
| Quartile 3 | 105/6284 | 1.14 (0.93-1.40) |  | 27/2151 | 0.81 (0.55-1.19) |  | 153/11126 | 0.90 (0.75-1.08) |  |
| Quartile 4 | 56/3762 | 1.02 (0.78-1.35) |  | 53/1878 | 1.72 (1.30-2.27) |  | 78/4846 | 1.08 (0.85-1.37) |  |
| **BMI>25kg/m^2^** | | | | | | | | | |
| 100g/day increment | 1684/114194 | 1.03 (1.01-1.06) | 0.005^*^ | 1684/114194 | 1.01 (0.99-1.03) | 0.562^*^ | 1684/114194 | 0.99 (0.96, 1.03) | 0.643^*^ |
| 0 | 1055/72944 | Reference |  | 1227/86509 | Reference |  | 902/57074 | Reference |  |
| Quartile 1 | 139/10184 | 0.97 (0.81-1.16) |  | 82/5931 | 0.93 (0.74-1.16) |  | 174/11146 | 1.03 (0.87-1.21) |  |
| Quartile 2 | 151/9455 | 1.14 (0.96-1.35) | 0.011 | 115/6878 | 1.10 (0.91-1.33) | 0.680 | 272/19824 | 0.98 (0.85-1.12) | 0.377 |
| Quartile 3 | 184/12841 | 1.02 (0.87-1.19) |  | 137/7146 | 1.21 (1.01-1.45) |  | 229/18003 | 0.92 (0.79-1.06) |  |
| Quartile 4 | 155/8770 | 1.27 (1.07-1.51) |  | 123/7730 | 0.96 (0.79-1.15) |  | 107/8147 | 0.97 (0.80-1.19) |  |

Note: All HRs were calculated by adjusting the following covariates: age, sex, Townsend deprivation index, education level, ethnicity, smoking status, alcohol drinking, IPAQ (International Physical Activity Questionnaire), total energy intake, type 2 diabetes, depression and anxiety. P for trend was calculated by using median value (82.5, 130, 250 and 500g/day) of each sugar-sweetened beverages Quartile, median value (82.5, 165, 330 and 660 g/day) of each artificially sweetened beverages Quartile, and median value (62.5, 125, 250 and 417 g/day) of each natural juice Quartile. *: Test for trend was performed by considering intake a continuous variable. P for interaction was 0.085 for sugar-sweetened beverages, 0.002 for artificially sweetened beverages and 0.926 for natural juice. IBS: irritable bowel syndrome; HR: hazard ratio; CI: confidence interval.
