# Supplementary material for: Sugar‐Sweetened Beverages, Artificially Sweetened Beverages and Sugar Forms With Long‐Term Risk of Irritable Bowel Syndrome: A Large‐Scale Prospective Cohort Study
Source: Food Sci Nutr. 2025 Mar 19;13(3):e70094. doi: 10.1002/fsn3.70094 (PMC11922681; doi:10.1002/fsn3.70094)
Supplement: Supplementary file 5 — Table S5. [file FSN3-13-e70094-s005.docx]

**Table S5.** **Risk of IBS associated with baseline sugar-sweetened beverages, artificially sweetened beverages and natural juice consumption stratified by smoking status.**

| **SSBs/ASBs/Natural juice consumption** | **Sugar-sweetened beverages** | | | **Artificially sweetened beverages** | | | **Natural juice** | | |
| --- | --- | --- | --- | --- | --- | --- | --- | --- | --- |
|  | **No. of IBS/**  **participants** | **HR (95%CI)** | **P for trend** | **No. of IBS/**  **participants** | **HR (95%CI)** | **P for trend** | **No. of IBS/ participants** | **HR (95%CI)** | **P for trend** |
| **Never smoking** | | | | | | | | | |
| 100g/day increment | 1488/101498 | 1.04 (1.02-1.07) | 0.002^*^ | 1488/101498 | 1.03 (1.01-1.05) | 0.015^*^ | 1488/101498 | 0.97 (0.93-1.01) | 0.096^*^ |
| 0 | 918/64398 | Reference |  | 1135/80801 | Reference |  | 741/46532 | Reference |  |
| Quartile 1 | 149/9985 | 1.05 (0.88-1.25) |  | 72/4895 | 1.02 (0.80-1.29) |  | 160/10488 | 0.98 (0.83-1.16) |  |
| Quartile 2 | 126/8654 | 1.05 (0.87-1.26) | 0.004 | 83/5305 | 1.09 (0.87-1.37) | 0.010 | 257/18760 | 0.92 (0.80-1.06) | 0.040 |
| Quartile 3 | 167/11233 | 1.08 (0.91-1.27) |  | 101/5272 | 1.30 (1.06-1.60) |  | 235/17774 | 0.90 (0.77-1.04) |  |
| Quartile 4 | 128/7228 | 1.33 (1.10-1.61) |  | 97/5225 | 1.22 (0.99-1.51) |  | 95/7944 | 0.84 (0.67-1.04) |  |
| **Previous/current smoking** | | | | | | | | | |
| 100g/day increment | 1191/76736 | 1.01 (0.98-1.04) | 0.577^*^ | 1191/76736 | 1.00 (0.98-1.03) | 0.768^*^ | 1191/76736 | 1.04 (1.00, 1.08) | 0.063^*^ |
| 0 | 770/50762 | Reference |  | 915/60520 | Reference |  | 637/39465 | Reference |  |
| Quartile 1 | 115/6905 | 1.13 (0.93-1.37) |  | 60/3731 | 1.01 (0.78-1.31) |  | 125/7685 | 1.04 (0.86-1.27) |  |
| Quartile 2 | 99/5874 | 1.13 (0.92-1.39) | 0.583 | 70/4073 | 1.05 (0.83-1.34) | 0.986 | 194/13188 | 1.03 (0.88-1.21) | 0.317 |
| Quartile 3 | 123/7891 | 1.04 (0.86-1.26) |  | 66/4031 | 0.96 (0.75-1.24) |  | 144/11344 | 0.90 (0.75-1.08) |  |
| Quartile 4 | 84/5304 | 1.04 (0.82-1.30) |  | 80/4381 | 1.01 (0.80-1.27) |  | 91/5054 | 1.29 (1.04-1.62) |  |

Note: All HRs were calculated by adjusting the following covariates: age, sex, Townsend deprivation index, education level, ethnicity, smoking status, alcohol drinking, IPAQ (International Physical Activity Questionnaire), total energy intake, type 2 diabetes, depression and anxiety. P for trend was calculated by using median value (82.5, 130, 250 and 500g/day) of each sugar-sweetened beverages Quartile, median value (82.5, 165, 330 and 660 g/day) of each artificially sweetened beverages Quartile, and median value (62.5, 125, 250 and 417 g/day) of each natural juice Quartile. *: Test for trend was performed by considering intake a continuous variable. P for interaction was 0.542 for sugar-sweetened beverages, 0.598 for artificially sweetened beverages and 0.114 for natural juice. IBS: irritable bowel syndrome; HR: hazard ratio; CI: confidence interval.
