# Supplementary material for: Sugar‐Sweetened Beverages, Artificially Sweetened Beverages and Sugar Forms With Long‐Term Risk of Irritable Bowel Syndrome: A Large‐Scale Prospective Cohort Study
Source: Food Sci Nutr. 2025 Mar 19;13(3):e70094. doi: 10.1002/fsn3.70094 (PMC11922681; doi:10.1002/fsn3.70094)
Supplement: Supplementary file 6 — Table S6. [file FSN3-13-e70094-s002.docx]

**Table S6.** **Risk of IBS associated with baseline sugar-sweetened beverages, artificially sweetened beverages and natural juice consumption stratified by alcohol drinking.**

| **SSBs/ASBs/Natural juice consumption** | **Sugar-sweetened beverages** | | | **Artificially sweetened beverages** | | | **Natural juice** | | |
| --- | --- | --- | --- | --- | --- | --- | --- | --- | --- |
|  | **No. of IBS/**  **participants** | **HR (95%CI)** | **P for trend** | **No. of IBS/**  **participants** | **HR (95%CI)** | **P for trend** | **No. of IBS/ participants** | **HR (95%CI)** | **P for trend** |
| **Never /Previous drinking** | | | | | | | | | |
| 100g/day increment | 243/10962 | 1.01 (0.96-1.07) | 0.582^*^ | 243/10962 | 0.98 (0.93-1.03) | 0.485^*^ | 243/10962 | 1.01 (0.93-1.09) | 0.803^*^ |
| 0 | 145/6428 | Reference |  | 189/8392 | Reference |  | 141/5947 | Reference |  |
| Quartile 1 | 13/964 | 0.61 (0.35-1.08) |  | 10/488 | 0.88 (0.46-1.66) |  | 21/961 | 0.94 (0.59-1.49) |  |
| Quartile 2 | 29/932 | 1.47 (0.99-2.20) | 0.932 | 13/536 | 1.09 (0.62-1.93) | 0.160 | 37/1532 | 1.15 (0.80-1.66) | 0.573 |
| Quartile 3 | 31/1463 | 1.01 (0.68-1.49) |  | 17/664 | 1.12 (0.67-1.84) |  | 26/1554 | 0.81 (0.53-1.23) |  |
| Quartile 4 | 25/1175 | 0.98 (0.63-1.52) |  | 14/882 | 0.62 (0.36-1.07) |  | 18/968 | 0.95 (0.57-1.56) |  |
| **Current drinking** | | | | | | | | | |
| 100g/day increment | 2442/167571 | 1.03 (1.01-1.05) | 0.010^*^ | 2442/167571 | 1.02 (1.00-1.04) | 0.020* | 2442/167571 | 1.00 (0.97-1.03) | 0.877^*^ |
| 0 | 1548/108915 | Reference |  | 1867/133153 | Reference |  | 1240/80204 | Reference |  |
| Quartile 1 | 252/15947 | 1.13 (0.99-1.29) |  | 123/8165 | 1.03 (0.86-1.23) |  | 264/17235 | 1.01 (0.89-1.16) |  |
| Quartile 2 | 195/13628 | 1.03 (0.89-1.20) | 0.011 | 139/8854 | 1.06 (0.90-1.27) | 0.013 | 414/30459 | 0.95 (0.85-1.07) | 0.433 |
| Quartile 3 | 260/17698 | 1.07 (0.94-1.22) |  | 150/8653 | 1.14 (0.97-1.35) |  | 356/27620 | 0.91 (0.81-1.03) |  |
| Quartile 4 | 187/11383 | 1.22 (1.04-1.42) |  | 163/8746 | 1.19 (1.01-1.40) |  | 168/12053 | 1.02 (0.87-1.20) |  |

Note: All HRs were calculated by adjusting the following covariates: age, sex, Townsend deprivation index, education level, ethnicity, smoking status, alcohol drinking, IPAQ (International Physical Activity Questionnaire), total energy intake, type 2 diabetes, depression and anxiety. P for trend was calculated by using median value (82.5, 130, 250 and 500g/day) of each sugar-sweetened beverages Quartile, median value (82.5, 165, 330 and 660 g/day) of each artificially sweetened beverages Quartile, and median value (62.5, 125, 250 and 417 g/day) of each natural juice Quartile. *: Test for trend was performed by considering intake a continuous variable. P for interaction was 0.071 for sugar-sweetened beverages, 0.372 for artificially sweetened beverages and 0.795 for natural juice. IBS: irritable bowel syndrome; HR: hazard ratio; CI: confidence interval.
