# Supplementary material for: Sugar‐Sweetened Beverages, Artificially Sweetened Beverages and Sugar Forms With Long‐Term Risk of Irritable Bowel Syndrome: A Large‐Scale Prospective Cohort Study
Source: Food Sci Nutr. 2025 Mar 19;13(3):e70094. doi: 10.1002/fsn3.70094 (PMC11922681; doi:10.1002/fsn3.70094)
Supplement: Supplementary file 7 — Table S7. [file FSN3-13-e70094-s004.docx]

**Table S7.** **Risk of IBS associated with baseline sugar-sweetened beverages, artificially sweetened beverages and natural juice consumption stratified by diet pattern.**

| **SSBs/ASBs/Natural juice consumption** | **Sugar-sweetened beverages** | | | **Artificially sweetened beverages** | | | **Natural juice** | | |
| --- | --- | --- | --- | --- | --- | --- | --- | --- | --- |
|  | **No. of IBS/**  **participants** | **HR (95%CI)** | **P for trend** | **No. of IBS/**  **participants** | **HR (95%CI)** | **P for trend** | **No. of IBS/ participants** | **HR (95%CI)** | **P for trend** |
| **Healthy** | | | | | | | | | |
| 100g/day increment | 2057/136027 | 1.02 (0.99-1.05) | 0.127^*^ | 2057/136027 | 1.03 (1.01-1.05) | 0.009^*^ | 2057/136027 | 1.00 (0.97-1.04) | 0.808^*^ |
| 0 | 1335/89946 | Reference |  | 1578/108847 | Reference |  | 1019/63187 | Reference |  |
| Quartile 1 | 206/13156 | 1.07 (0.92-1.23) |  | 99/6615 | 1.00 (0.81-1.22) |  | 223/14183 | 1.01 (0.87-1.17) |  |
| Quartile 2 | 177/10939 | 1.11 (0.95-1.30) | 0.151 | 124/7038 | 1.17 (0.98-1.41) | 0.015 | 365/25068 | 0.99 (0.88-1.12) | 0.455 |
| Quartile 3 | 208/13671 | 1.06 (0.91-1.22) |  | 130/6797 | 1.24 (1.04-1.49) |  | 300/23124 | 0.89 (0.78-1.02) |  |
| Quartile 4 | 131/8315 | 1.11 (0.93-1.34) |  | 126/6730 | 1.16 (0.96-1.39) |  | 150/10465 | 1.03 (0.86-1.22) |  |
| **Unhealthy** | | | | | | | | | |
| 100g/day increment | 633/42684 | 1.03 (1.00-1.07) | 0.041^*^ | 633/42684 | 0.99 (0.96-1.02) | 0.587 | 633/42684 | 0.99 (0.93-1.05) | 0.680^*^ |
| 0 | 362/25508 | Reference |  | 481/32841 | Reference |  | 365/23068 | Reference |  |
| Quartile 1 | 59/3769 | 1.14 (0.86-1.50) |  | 34/2044 | 1.09 (0.77-1.54) |  | 62/4021 | 1.02 (0.78-1.34) |  |
| Quartile 2 | 48/3639 | 0.96 (0.71-1.30) | 0.046 | 29/2363 | 0.79 (0.54-1.14) | 0.642 | 87/6950 | 0.89 (0.71-1.13) | 0.728 |
| Quartile 3 | 83/5510 | 1.06 (0.84-1.35) |  | 37/2525 | 0.86 (0.61-1.20) |  | 83/6074 | 0.98 (0.77-1.24) |  |
| Quartile 4 | 81/4258 | 1.31 (1.02-1.68) |  | 52/2911 | 0.99 (0.74-1.33) |  | 36/2571 | 0.98 (0.69-1.38) |  |

Note: All HRs were calculated by adjusting the following covariates: age, sex, Townsend deprivation index, education level, ethnicity, smoking status, alcohol drinking, IPAQ (International Physical Activity Questionnaire), total energy intake, type 2 diabetes, depression and anxiety. P for trend was calculated by using median value (82.5, 130, 250 and 500g/day) of each sugar-sweetened beverages Quartile, median value (82.5, 165, 330 and 660 g/day) of each artificially sweetened beverages Quartile, and median value (62.5, 125, 250 and 417 g/day) of each natural juice Quartile. *: Test for trend was performed by considering intake a continuous variable. P for interaction was 0.542 for sugar-sweetened beverages, 0.218 for artificially sweetened beverages and 0.819 for natural juice. IBS: irritable bowel syndrome; HR: hazard ratio; CI: confidence interval.
