# Supplementary material for: Sugar‐Sweetened Beverages, Artificially Sweetened Beverages and Sugar Forms With Long‐Term Risk of Irritable Bowel Syndrome: A Large‐Scale Prospective Cohort Study
Source: Food Sci Nutr. 2025 Mar 19;13(3):e70094. doi: 10.1002/fsn3.70094 (PMC11922681; doi:10.1002/fsn3.70094)
Supplement: Supplementary file 8 — Table S8. [file FSN3-13-e70094-s011.docx]

**Table S8.** **Results of IBS risk associated with baseline artificially sweetened beverages consumption.**

| **Variables** | **Category** | **HR (95%CI)** | **P value** |
| --- | --- | --- | --- |
| ASB consumption | 0 | Reference |  |
|  | Quartile 1 | 1.01 (0.85, 1.21) | 0.869 |
|  | Quartile 2 | 1.07 (0.91, 1.26) | 0.413 |
|  | Quartile 3 | 1.14 (0.97, 1.33) | 0.115 |
|  | Quartile 4 | 1.12 (0.95, 1.31) | 0.168 |
| Age | Per year | 1.00 (0.99, 1.00) | 0.403 |
| Sex | Male | Reference |  |
|  | Female | 1.84 (1.69, 2.01) | <0.001 |
| BMI | Normal | Reference |  |
|  | Underweight | 1.02 (0.62, 1.67) | 0.933 |
|  | Overweight | 0.98 (0.89, 1.07) | 0.613 |
|  | Obesity | 0.98 (0.88, 1.09) | 0.684 |
| TDI | Quartile 1 | Reference |  |
|  | Quartile 2 | 0.98 (0.88, 1.10) | 0.775 |
|  | Quartile 3 | 1.01 (0.90, 1.12) | 0.913 |
|  | Quartile 4 | 1.06 (0.95, 1.18) | 0.328 |
| Education | Non-university | Reference |  |
|  | University | 0.77 (0.71, 0.84) | <0.001 |
| Ethnicity | Non-White | Reference |  |
|  | White | 1.17 (0.96, 1.42) | 0.126 |
| Alcohol drinking | Never | Reference |  |
|  | Previous | 1.76 (1.35, 2.28) | <0.001 |
|  | Current | 1.00 (0.81, 1.24) | 0.981 |
| Smoking | Never | Reference |  |
|  | Previous | 1.08 (0.99, 1.17) | 0.068 |
|  | Current | 1.05 (0.91, 1.21) | 0.528 |
| IPAQ | Low | Reference |  |
|  | Moderate | 0.83 (0.74, 0.93) | 0.001 |
|  | High | 0.80 (0.71, 0.90) | <0.001 |
| Type 2 Diabetes | No | Reference |  |
|  | Yes | 1.44 (1.14, 1.83) | 0.002 |
| Depression |  | Reference |  |
|  |  | 1.80 (1.61, 2.02) | <0.001 |
| Anxiety |  | Reference |  |
|  |  | 1.84 (1.58, 2.14) | <0.001 |
| Total energy intake | Per 1 kJ | 1.00 (1.00, 1.00) | 0.304 |

Note: All HRs were calculated by adjusting the following covariates in addition to ASB quartiles as exposure: age, sex, BMI, Townsend deprivation index, education level, ethnicity, smoking status, alcohol drinking, IPAQ (International Physical Activity Questionnaire), total energy intake, type 2 diabetes, depression and anxiety. IBS: irritable bowel syndrome; HR: hazard ratio; CI: confidence interval.
