# Supplementary material for: Sugar‐Sweetened Beverages, Artificially Sweetened Beverages and Sugar Forms With Long‐Term Risk of Irritable Bowel Syndrome: A Large‐Scale Prospective Cohort Study
Source: Food Sci Nutr. 2025 Mar 19;13(3):e70094. doi: 10.1002/fsn3.70094 (PMC11922681; doi:10.1002/fsn3.70094)
Supplement: Supplementary file 10 — Table S10. [file FSN3-13-e70094-s006.docx]

**Table S10.** **Sensitivity analysis regarding risk of IBS associated with baseline sugar-sweetened beverages, artificially sweetened beverages and natural juice consumption.**

| **SSBs/ASBs/Fruit juice consumption** | **Sugar-sweetened beverages** | | | **Artificially sweetened beverages** | | | **Natural juice** | | |
| --- | --- | --- | --- | --- | --- | --- | --- | --- | --- |
|  | **No. of IBS/**  **participants** | **HR (95%CI)** | **P for trend** | **No. of IBS/**  **participants** | **HR (95%CI)** | **P for trend** | **No. of IBS/ participants** | **HR (95%CI)** | **P for trend** |
| Sensitivity analysis 1: excluding IBS participants diagnosed within 1 year after 24-hour dietary recall questionnaire (N=178434) | | | | | | | | | |
| 100g/day increment | 2413/178434 | 1.03 (1.01-1.05) | 0.012^*^ | 2413/178434 | 1.01 (1.00-1.03) | 0.147^*^ | 2413/178434 | 1.00 (0.97-1.03) | 0.938^*^ |
| 0 | 1515/115272 | Reference |  | 1859/141488 | Reference |  | 1242/86113 | Reference |  |
| Quartile 1 | 240/16900 | 1.10 (0.96-1.26) |  | 118/8644 | 1.00 (0.83-1.20) |  | 252/18171 | 0.99 (0.87-1.14) |  |
| Quartile 2 | 208/14561 | 1.12 (0.97-1.29) | 0.018 | 127/9375 | 0.99 (0.82-1.18) | 0.148 | 409/31975 | 0.98 (0.87-1.09) | 0.445 |
| Quartile 3 | 264/19154 | 1.08 (0.95-1.24) |  | 154/9309 | 1.16 (0.99-1.38) |  | 344/29159 | 0.91 (0.81-1.03) |  |
| Quartile 4 | 186/12547 | 1.18 (1.01-1.38) |  | 157/9618 | 1.08 (0.92-1.28) |  | 166/13016 | 1.01 (0.86-1.19) |  |
| Sensitivity analysis 2: excluding IBS participants diagnosed within 2 years after 24-hour dietary recall questionnaire (N=178185) | | | | | | | | | |
| 100g/day increment | 2164/178185 | 1.02 (1.00-1.05) | 0.036* | 2164/178185 | 1.01 (0.99-1.03) | 0.311^*^ | 2164/178185 | 1.00 (0.97-1.03) | 0.905^*^ |
| 0 | 1357/115114 | Reference |  | 1674/141303 | Reference |  | 1114/85985 | Reference |  |
| Quartile 1 | 219/16879 | 1.12 (0.97-1.29) |  | 102/8628 | 0.96 (0.78-1.17) |  | 230/18149 | 1.01 (0.88-1.17) |  |
| Quartile 2 | 193/14546 | 1.16 (1.00-1.35) | 0.045 | 114/9362 | 0.98 (0.81-1.18) | 0.331 | 364/31930 | 0.98 (0.87-1.10) | 0.417 |
| Quartile 3 | 233/19123 | 1.07 (0.93-1.23) |  | 138/9293 | 1.15 (0.97-1.37) |  | 312/29127 | 0.93 (0.82-1.05) |  |
| Quartile 4 | 162/12523 | 1.15 (0.98-1.36) |  | 136/9599 | 1.05 (0.88-1.25) |  | 147/12994 | 0.99 (0.83-1.18) |  |
| Sensitivity analysis 3: mutually adjusted sugar-sweetened beverages and fruit juice consumption (N=178711) | | | | | | | | | |
| 100g/day increment | 2690/178711 | 1.03 (1.01-1.05) | 0.010^*^ | 2690/178711 | 1.02 (1.00-1.03) | 0.060^*^ | 2690/178711 | 1.00 (0.97-1.03) | 0.925^*^ |
| 0 | 1697/115454 | Reference |  | 2059/141688 | Reference |  | 1384/86255 | Reference |  |
| Quartile 1 | 265/16925 | 1.08 (0.94-1.23) |  | 133/8659 | 1.00 (0.84-1.20) |  | 285/18204 | 1.00 (0.87-1.13) |  |
| Quartile 2 | 225/14578 | 1.08 (0.94-1.24) | 0.018 | 153/9401 | 1.07 (0.91-1.26) | 0.075 | 452/32018 | 0.96 (0.86-1.07) | 0.347 |
| Quartile 3 | 291/19181 | 1.06 (0.94-1.20) |  | 167/9322 | 1.12 (0.96-1.32) |  | 383/29198 | 0.91 (0.81-1.02) |  |
| Quartile 4 | 212/12573 | 1.18 (1.02-1.37) |  | 178/9641 | 1.11 (0.95-1.30) |  | 186/13036 | 1.01 (0.86-1.18) |  |
| Sensitivity analysis 4: without adjusting total energy intake (N=178711) | | | | | | | | | |
| 100g/day increment | 2690/178711 | 1.02 (1.00-1.04) | 0.018^*^ | 2690/178711 | 1.02 (1.00-1.03) | 0.056^*^ | 2690/178711 | 1.00 (0.97-1.02) | 0.834^*^ |
| 0 | 1697/115454 | Reference |  | 2059/141688 | Reference |  | 1384/86255 | Reference |  |
| Quartile 1 | 265/16925 | 1.08 (0.94-1.22) |  | 133/8659 | 1.02 (0.85-1.21) |  | 285/18204 | 1.01 (0.89-1.14) |  |
| Quartile 2 | 225/14578 | 1.07 (0.93-1.23) | 0.028 | 153/9401 | 1.07 (0.91-1.26) | 0.062 | 452/32018 | 0.96 (0.87-1.07) | 0.297 |
| Quartile 3 | 291/19181 | 1.05 (0.93-1.19) |  | 167/9322 | 1.14 (0.97-1.33) |  | 383/29198 | 0.90 (0.80-1.01) |  |
| Quartile 4 | 212/12573 | 1.17 (1.01-1.35) |  | 178/9641 | 1.12 (0.95-1.30) |  | 186/13036 | 1.00 (0.86-1.17) |  |
| Sensitivity analysis 5: competing risk model (N=178711, No. of competing events=8480) | | | | | | | | | |
| 100g/day increment | 2690/178711 | 1.03(1.01-1.05) | 0.012^*^ | 2690/178711 | 1.02 (1.00-1.03) | 0.057^*^ | 2690/178711 | 1.00 (0.97-1.03) | 0.949^*^ |
| 0 | 1697/115454 | Reference |  | 2059/141688 | Reference |  | 1384/86255 | Reference |  |
| Quartile 1 | 265/16925 | 1.08 (0.95-1.23) |  | 133/8659 | 1.02 (0.85-1.21) |  | 285/18204 | 1.01 (0.89-1.15) |  |
| Quartile 2 | 225/14578 | 1.08 (0.94-1.24) | 0.020 | 153/9401 | 1.07 (0.91-1.27) | 0.069 | 452/32018 | 0.97 (0.87-1.08) | 0.366 |
| Quartile 3 | 291/19181 | 1.06 (0.93-1.20) |  | 167/9322 | 1.13 (0.97-1.33) |  | 383/29198 | 0.91 (0.81-1.02) |  |
| Quartile 4 | 212/12573 | 1.18 (1.02-1.37) |  | 178/9641 | 1.11 (0.95-1.31) |  | 186/13036 | 1.01 (0.87-1.18) |  |
| Sensitivity analysis 6: additionally adjusted diet pattern (N=179402) | | | | | | | | | |
| 100g/day increment | 2690/178711 | 1.03 (1.01-1.05) | 0.012^*^ | 2690/178711 | 1.02 (1.00-1.03) | 0.059^*^ | 2690/178711 | 1.00 (0.97-1.03) | 0.996^*^ |
| 0 | 1697/115454 | Reference |  | 2059/141688 | Reference |  | 1384/86255 | Reference |  |
| Quartile 1 | 265/16925 | 1.08 (0.95-1.23) |  | 133/8659 | 1.01 (0.85-1.21) |  | 285/18204 | 1.01 (0.89-1.15) |  |
| Quartile 2 | 225/14578 | 1.08 (0.94-1.24) | 0.019 | 153/9401 | 1.07 (0.91-1.26) | 0.066 | 452/32018 | 0.97 (0.87-1.08) | 0.394 |
| Quartile 3 | 291/19181 | 1.06 (0.93-1.20) |  | 167/9322 | 1.14 (0.97-1.33) |  | 383/29198 | 0.91 (0.81-1.02) |  |
| Quartile 4 | 212/12573 | 1.18 (1.02-1.37) |  | 178/9641 | 1.11 (0.95-1.30) |  | 186/13036 | 1.02 (0.87-1.19) |  |
| Sensitivity analysis 7: using quintiles of beverages consumption (N=179402)^$^ | | | | | | | | | |
| 0 | 1697/115454 | Reference |  | 2059/141688 | Reference |  | 1384/86255 | Reference |  |
| Quintile 1 | 162/10537 | 1.06 (0.90-1.25) |  | 67/4962 | 0.89 (0.70-1.14) |  | 285/18204 | 1.01 (0.89-1.15) |  |
| Quintile 2 | 228/14812 | 1.06 (0.93-1.22) | 0.014 | 161/9805 | 1.09 (0.93-1.28) | 0.036 | 313/21643 | 0.99 (0.87-1.12) | 0.382 |
| Quintile 3 | 165/10464 | 1.11 (0.95-1.31) |  | 58/3293 | 1.15 (0.88-1.49) |  | 174/13698 | 0.88 (0.75-1.04) |  |
| Quintile 4 | 226/14871 | 1.06(0.92-1.22) |  | 184/10462 | 1.12 (0.96-1.30) |  | 348/25875 | 0.93 (0.82-1.04) |  |
| Quintile 5 | 212/12573 | 1.19 (1.03-1.37) |  | 161/8501 | 1.14 (0.97-1.34) |  | 186/13036 | 1.01 (0.87-1.18) |  |
| Sensitivity analysis 8: excluding participants with total energy intake >4,200 kcal or <800 kcal for male and >3500 kcal or < 500 kcal for female (N=177085) | | | | | | | | | |
| 100g/day increment | 2664/177085 | 1.02 (1.00-1.05) | 0.018 | 2664/177085 | 1.02 (1.00-1.03) | 0.060 | 2664/177085 | 1.00 (0.97-1.03) | 0.857 |
| 0 | 1685/114518 | Reference |  | 2043/140418 | Reference |  | 1372/85408 | Reference |  |
| Quartile 1 | 262/16870 | 1.07 (0.94-1.22) |  | 131/8628 | 1.00 (0.84-1.20) |  | 285/18148 | 1.01 (0.89-1.15) |  |
| Quartile 2 | 224/14478 | 1.08 (0.94-1.24) | 0.024 | 152/9341 | 1.07 (0.91-1.26) | 0.080 | 449/31815 | 0.97 (0.87-1.08) | 0.259 |
| Quartile 3 | 287/18936 | 1.06 (0.93-1.20) |  | 163/9193 | 1.12 (0.96-1.32) |  | 378/28890 | 0.90 (0.80-1.01) |  |
| Quartile 4 | 206/12283 | 1.18 (1.01-1.36) |  | 175/9505 | 1.11 (0.95-1.30) |  | 180/12824 | 0.99 (0.85-1.16) |  |

Note: All HRs except for sensitivity 4 were calculated by adjusting the following covariates: age, sex, BMI, Townsend deprivation index, education level, ethnicity, smoking status, alcohol drinking, IPAQ (International Physical Activity Questionnaire), total energy intake, type 2 diabetes, depression and anxiety. P for trend was calculated by using median value (82.5, 130, 250 and 500g/day) of each sugar-sweetened beverages Quartile, median value (82.5, 165, 330 and 660 g/day) of each artificially sweetened beverages Quartile, and median value (62.5, 125, 250 and 417 g/day) of each natural juice Quartile. *: Test for trend was performed by considering intake a continuous variable. $: P for trend was calculated by using median value (62.5, 125, 167, 260 and 500g/day) of each sugar-sweetened beverages Quintile, median value (82.5, 165, 220, 330 and 660 g/day) of each artificially sweetened beverages Quintile, and median value (62.5, 125, 188, 250 and 417 g/day) of each natural juice Quintile. IBS: irritable bowel syndrome; HR: hazard ratio; CI: confidence interval.
