# Supplementary figures and images for: Sugar‐Sweetened Beverages, Artificially Sweetened Beverages and Sugar Forms With Long‐Term Risk of Irritable Bowel Syndrome: A Large‐Scale Prospective Cohort Study
Source: Food Sci Nutr. 2025 Mar 19;13(3):e70094. doi: 10.1002/fsn3.70094 (PMC11922681; doi:10.1002/fsn3.70094)

**Figure S1. Flowchart of the study population.**


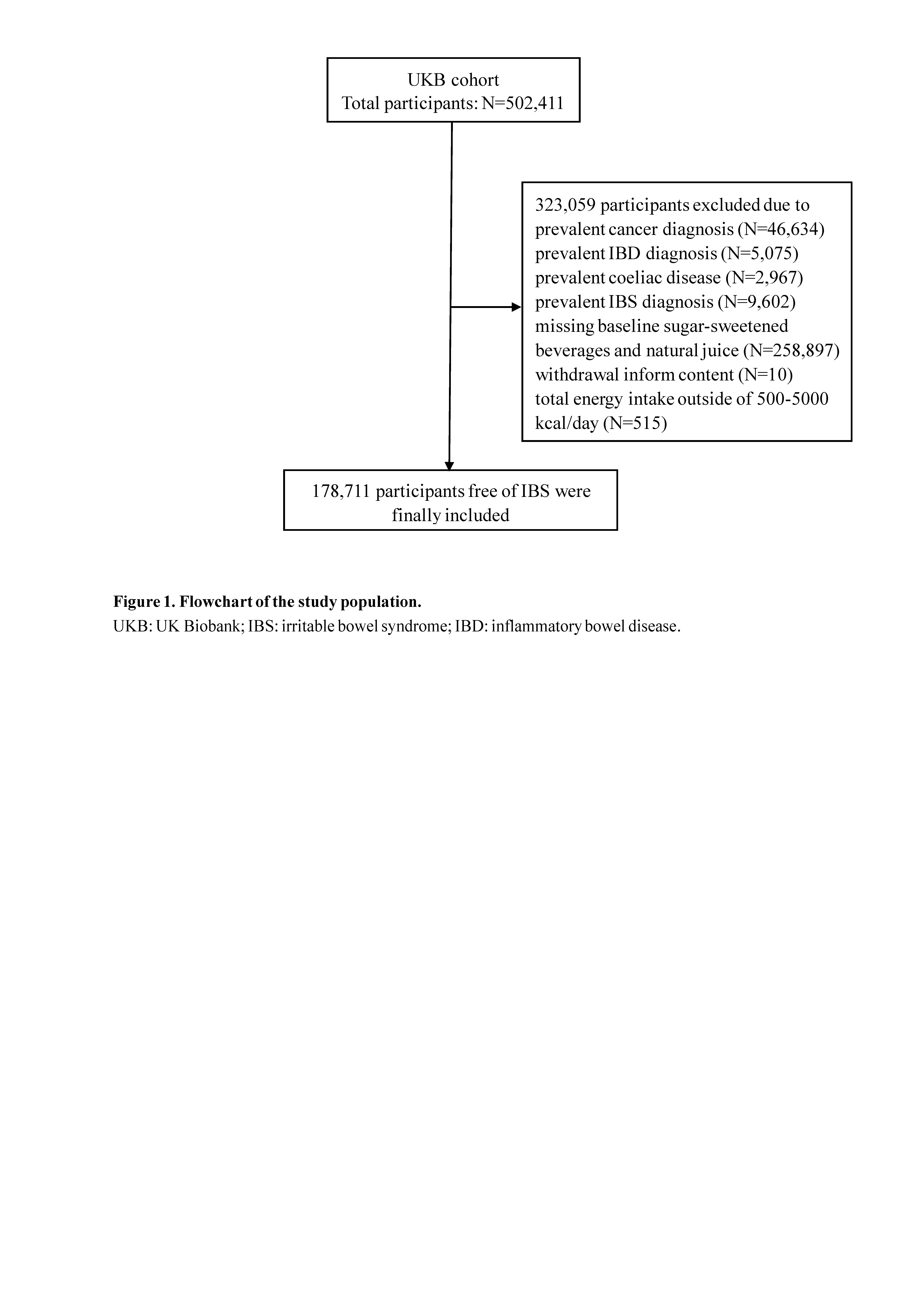

Supplement: Supplementary file 11 — Table S11. [file FSN3-13-e70094-s010.docx]
